# Phytochemistry, Pharmacology and Medicinal Uses of Plants of the Genus *Salix*: An Updated Review

**DOI:** 10.3389/fphar.2021.593856

**Published:** 2021-02-12

**Authors:** Nora Tawfeek, Mona F. Mahmoud, Dalia I Hamdan, Mansour Sobeh, Nawaal Farrag, Michael Wink, Assem M. El-Shazly

**Affiliations:** ^1^ Institute of Pharmacy and Molecular Biotechnology, Heidelberg University, Heidelberg, Germany; ^2^ Department of Pharmacognosy, Faculty of Pharmacy, Zagazig University, Zagazig, Egypt; ^3^ Department of Pharmacology and Toxicology, Faculty of Pharmacy, Zagazig University, Zagazig, Egypt; ^4^ Department of Pharmacognosy, Faculty of Pharmacy, Menoufia University, Shibin Elkom, Egypt; ^5^ AgroBioSciences Research Division, Mohammed VI Polytechnic University, Ben-Guerir, Morocco

**Keywords:** salix, phytochemistry, pharmacology, medicinal and traditional uses, inflammation

## Abstract

The Willows (genus *Salix*), with more than 330–500 species and 200 hybrids, are trees, shrubs or prostrate plants that are widely distributed in Africa, North America, Europe, and Asia. The genus is traditionally used in folk medicine and represents a valuable source of biologically active compounds among them salicin, a prodrug for salicylic acid. Altogether, 322 secondary metabolites were characterized in the genus including flavonoids 94) (flavonols, flavones, flavanones, isoflavones, flavan-3-ols (catechins and procyanidins), chalcones, dihydrochalcone, anthocyanins, dihydroflavonols), phenolic glycosides (76), organic acids (28), and non-phenolic glycosides (17), sterols and terpenes (17), simple phenolics 13) and lignans 7) in addition to volatiles and fatty acids (69). Furthermore, willows exert analgesic, anti-inflammatory, antioxidant, anticancer, cytotoxic, antidiabetic, antimicrobial, antiobesity, neuroprotective and hepatoprotective activities. The current review provides an updated summary of the importance of willows, their chemical composition and pharmacological activities.

## Introduction

Salicaceae (the Willow and Poplar family) traditionally includes the genera *Populus* (poplar) and *Salix* (willow), which are common in Northern temperate regions, and are amentiferous (bearing catkins) (Isebrands and Richardson, 2014). Presently, the Salicaceae have been enlarged to contain most tropical members of Flacourtiaceae, which do not produce catkin (Thadeo et al., 2014). Thus, the family Salicaceae now comprises about 56 genera and 1,220 species (Christenhusz and Byng, 2016).

The members of Salicaceae are fast growing trees or shrubs (Isebrands and Richardson, 2014). They are used for many economic purposes as production of timber, paper, fences, shelter, snowshoes, arrow shafts, fish traps, whistles, nets, rope, as a biomass fuel (a source of renewable energy), for ornamental, architectural and horticulture uses. Also, they are used for environmental enhancement through soil erosion control (Kuzovkina and Vietto, 2014). Willow twigs are elastic and were used to interweave baskets, for caning, and to manufacture woven fences and other lattices (Isebrands and Richardson, 2014).

The genus *Salix* (the willow) includes 330–500 species and more than 200 hybrids (Isebrands and Richardson, 2014), which are most widely distributed in the Northern hemisphere with a limited number of species occur in the Southern hemisphere (Zhen-Fu, 1987). *Salix* species are widely distributed in Africa, North America, Europe, and Asia (Argus, 2007). *Salix* species are fast growing trees, shrubs or prostrate plants; they can withstand a wide range of different weathers more than *Populus* species, as they grow in temperate, subtropic and tropic regions (Isebrands and Richardson, 2014).

### Taxonomy

General morphological characters of genus *Salix* were reported (Argus, 2006; Lauron-Moreau, et al., 2015). Willows are 6–10 m high trees or shrubs with spirally arranged, sometimes silvery, oblong leaves. The latter is commonly hairy on the underside and often turn black when drying. Leaves are simple, petiolate showing different shapes of lamina (oblong, linear, ovate, obovate or round), stipulate with linear to rounded stipules and with entire, serrate or dentate margin. Their arrangement is mostly alternate or rarely opposite (Lauron-Moreau, et al., 2015). The flowers are catkins, dioecious, with nectaries (glands) instead of perianth and they have bracts, which are pale or black, pubescent or glabrate, constant in male flowers and deciduous in female ones. The flowers blossom in spring, generally prior the leaves (Mabberley 2008). The male catkins have mostly two stamens, more prominent yellow, with few species having 3–12 stamens while the female catkins are greenish, have single pistil with single ovary, style, two-lobed stigma and 2 to 42 ovules per each ovary (Mabberley 2008). The nectar of flowering Willow is the first food source for bees in spring. The seeds are small, with limited longevity, fine hairy coat enabling their spread by wind and they germinate after few days of exposure to moistured surfaces (Mabberley 2008). Recently, the taxonomy of neotropical Salicaceae (formerly Flacourtiaceae) is difficult, as they show very different morphology and exhibit numerous characteristics in common with several other families. The neotropical Salicaceae and Salicaceae displayed similar characters such as the presence of salicoid leaf teeth, collateral and arch-shaped vascular system at the midrib, abundance of crystals, brachyparacytic stomata, secondary growth of the petiole and sclerenchyma accompanying the bundles (Thadeo et al., 2014).

### Phytochemistry

Different phytoconstituents or secondary metabolites of the genus *Salix* as flavonoids, glycosides (phenolic and non-phenolic glycosides), procyanidins, organic acids and their derivatives, simple phenolics, sterols and terpenes, lignans, volatiles and fatty acids were reported (Supplementary Tables S1–S7, included in Supplementary materials). *Salix* leaves mainly contain flavonoids, phenolic acids, their derivatives, and phenolic glycosides, while stem bark mainly contains procyanidins.

### Flavonoids

**FIGURE 1:**
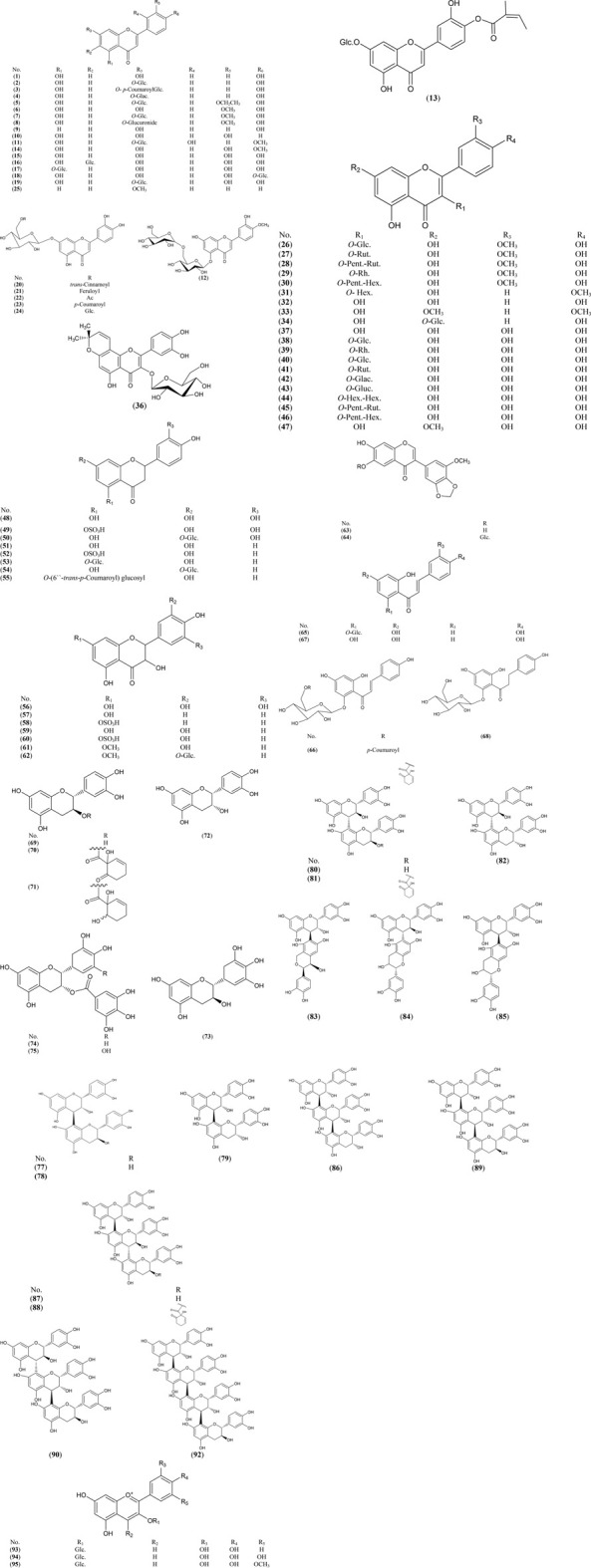
Structures of reported flavonoids from the genus *Salix.*

*Salix* contains a wide variety of flavonoids, which are distinctive for each species, as flavones, flavonols, flavanones, dihydroflavonols, isoflavones, chalcones, dihydrochalcones, flavan-3-ols and anthocyanins (Nasudari et al., 1972; Pobł ocka-Olech and Krauze-Shao et al., 1989; Du et al., 2004; Zeid, 2006; Jü rgenliemk et al., 2007; Baranowska, 2008; Freischmidt et al., 2010; Li et al., 2013). Data are summarized in Supplementary Table S1 and the structures are presented in Figure 1.

The highest numbers of different classes of flavonoids (A-E) were detected in leaves and rarely in roots. The flavones as apigenin and its glycosides (1, 2, 4, 5) are major constituents of *S. acutifolia* Willd. leaves (Shelyuto and Bondarenko, 1985), *S. matsudana* Koidz. leaves (Han et al., 2003a) and *S. babylonica* L. leaves and roots (Khatoon et al., 1988; Singh et al., 2017). Whereas, chrysoeriol (6), its 7-*O*-D-glucoside 7) and 7-*O*-glucuronide 8) are major constituents of *S. babylonica* L. (Liu et al., 2008), *S. matsudana* Koidz. leaves (Han et al., 2003b) and *S. subserrata* Willd. leaves (Tawfeek et al., 2019), respectively. Compounds (12, 14) were reported in *S. denticulate* leav*es* (Rawat et al., 2009; Semwal et al., 2011). *S. gilgiana* Seemen. leaves were characterized by the accumulation of acylated luteolin glucosides (19–23) (Mizuno et al., 1987). Compounds (25, 35) are chemical markers for *S. matsudana* Koidz. leaves (Li et al., 2008). Kaempferol 32) and its 7,4ˋ-dimethyl derivative 33) were found to be most prominent constituents in *S. bordensis* Turcz. (Zhao et al., 2014). Also, kaempferol-7-*O*-glucoside 34) is a major compound in *S. babylonica* L. leaves and roots (Khatoon et al., 1988; Singh et al., 2017).

Angeloxyflavone 13) and isoflavones (63, 64) are chemical markers for *S. cheilophila* C. K. Schneid. twigs (Shen et al., 2008). *S. integra × S. suchowensis* young stem was characterized by the accumulation of sulfated flavanones and dihydroflavonol as compounds (49, 52, 58, 60). Compound 11) was reported in the erial parts of *S. denticulate* Andersson.

The highest number of chalcones, catechins, procyanidins and anthocyanins were detected in the bark of willows*.* The bark of *S. daphnoides* Vill.*, S. elbursensis* Boiss.*, S. acutifolia* Willd. and *S. rubra* Huds. were characterized by the accumulation of chalcones (65–67) (Kompantsev, 1969; Kompantsev and Shinkarenko, 1975; Vinokurov, 1979; Zapesochnaya et al., 2002; Krauze-Baranowska et al., 2013). Catechin 69) and its derivatives (70, 71), epicatechin (72), procyanidin B1 77) and its derivative (78), procyanidin B3 (80) and its derivative (81), procyanidins B6 (84), B7 85) and trimeric procyanidins (87–89) were found to be major constituents of *S. sieboldiana* Blume bark (Hsu et al., 1985). Also, procyanidins (77, 79, 80, 82, 83, 85, 86, 89, 90, 92) are major constituents of *S. daphnoides* Vill. bark (Wiesneth, 2019). Anthocyanins (93–95) were detected in the bark of *S. purpurea* L., *S. daphnoides* Vill.*, S. alba* L.*, S. phylicifolia* L.*, S. nigricans* Sm.*, S. calodendron* Wimm. *and S. viminalis* L.*, S. triandra* L. and *S. amygdalina* L. (Bridle et al., 1970; Bridle et al., 1973).

### Phenolic Glycosides

**FIGURE 2:**
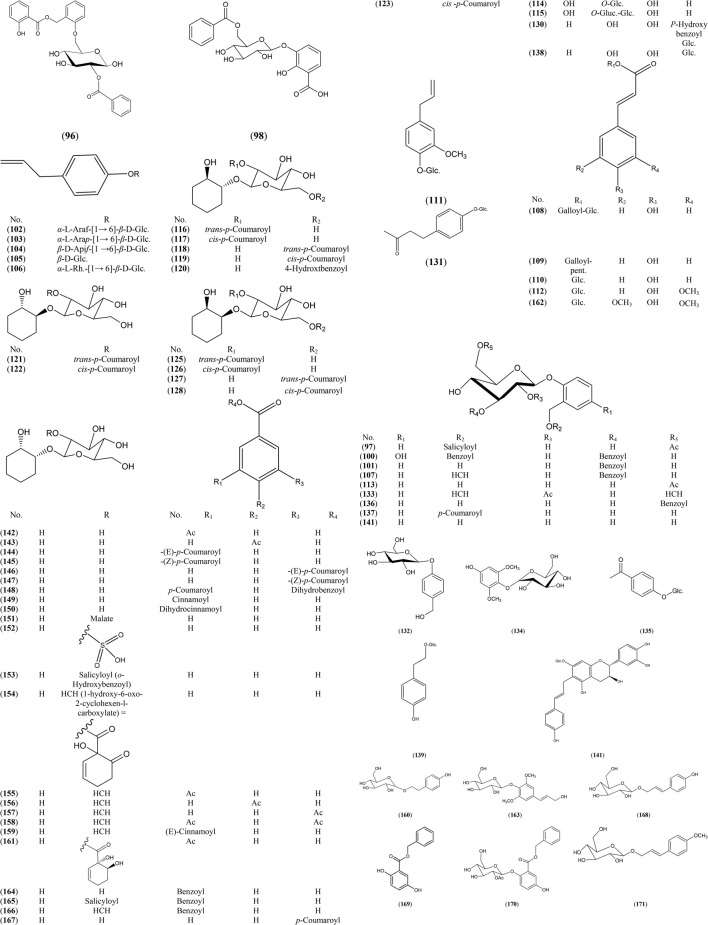
Structures of reported phenolic glycosides from genus *Salix.*

Glycosides are major secondary metabolites in Salicaceae (Binns et al., 1968; Kompantsev and Shinkarenko, 1973; Kompantsev et al., 1974; Nichols‐Orians et al., 1992; Fernandes et al., 2009). Phenolic glycosides represent up to 30% of dry plant mass. They are classified into two main classes: Salicin derived glycosides (salicinoids) and other phenolic glycosides as glycosylated phenylpropanoids, phenylethanoids and benzenoids and glycosylated salicylic acid derivatives. Salicinoids, which are considered as taxonomic markers for genus *Salix*, are derivatives of salicin, produced by esterification of one or more hydroxyl groups of salicyl alcohol or glucose moieties, mainly 2ˋ and/or 6′ of glucose, with organic acids as acetic, benzoic and 1-hydroxy-6-oxocyclohex-2-en-1-carboxylic (HCH) acids. The phenolic glycosides isolated and/or identified from genus *Salix* are presented in Supplementary Table S2 and Figure 2.

The highest number of phenolic glycosides were reported in *Salix* leaves, followed by twigs, stems and bark. Salicin (141), tremuloidin (164), tremulacin 166) were found to be the major constituents in *S. Acutifolia* Willd. juvenile stem and bark (Zapesochnaya et al., 2002; Wu et al., 2016), *S. chaenomeloides* Kimura leaves (Mizuno et al., 1991), *S. glandulosa* Seemen. twigs (Kim et al., 2015) and *S. tetrasperma* Roxb. leaves (El-Shazly et al., 2012).

Some phenolic glycosides were identified as taxonomic markers for different *Salix* species. Acmophyllin A 96) and acmophyllin B 97) identified as taxonomic marker for *S. acmophylla* Boiss. leaves (Shah et al., 2016). Chaenomeloidin (101), cochinchiside A (107), lasiandrin (133), leonuriside A (134), salicin-7-sulfate 152) identified as taxonomic markers for *S. chaenomeloides* Kimura leaves (Mizuno et al., 1991), *S. glandulosa* Seemen. twigs (Kim et al., 2015), *S. lasiandra* leaves and twigs (Reichardt et al., 1992), *S. matsudana* Koidz. leaves (Li et al., 2008) and *S. koriyanagi* Kimura. Stems (Noleto-Dias et al., 2018), respectively. Sachaliside 1 139) and sachaliside 2 (140) were identified as taxonomic markers for *S. sachalinensis* F. Schmidt (Mizuno et al., 1990).

Some *Salix* species were characterized by accumulation of 1,2-cyclohexanediol glycosides. Compounds (116–128) were detected in *S. glandulosa* Seemen. twigs (Kim et al., 2014). Also, acutifoliside, a benzoic acid derivative 98) was a chemical marker for *S. acutifolia* Willd. juvenile stem (Wu et al., 2016).

### Non-Phenolic Glycosides

**FIGURE 3:**
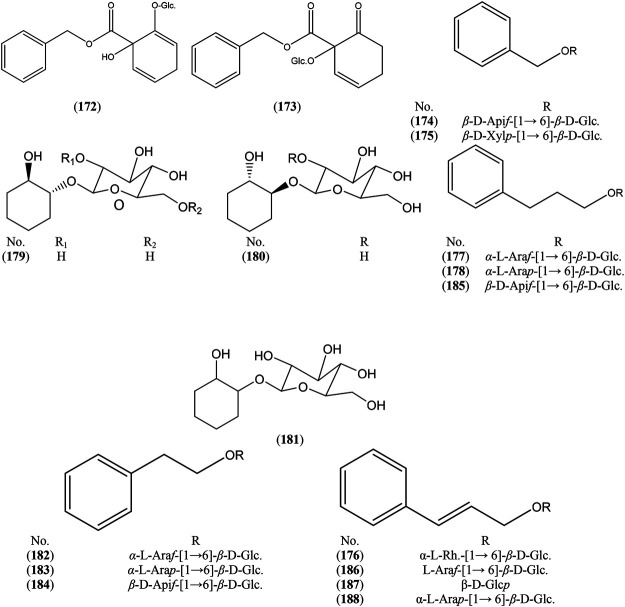
Structures of reported non-phenolic glycosides from genus *Salix.*

Non-phenolic glycosides (172, 173, 174, 175, 176, 182–188) were found to be the major constituents in *S. triandra* L. *x dasyclados* Wimmer Wood (Noleto-Dias et al., 2019). Also, compounds (170, 171) are the major constituents in *S. arbusculoides* Andersson twigs (Evans et al., 1995). Some *Salix* species were characterized by accumulation of 1,2-cyclohexanediol glycosides. Compounds (177, 180) were detected in *S. glandulosa* Seemen. twigs (Kim et al., 2014) and grandidentin 181) was reported in *S. purpurea* L. bark (Pearl and Darling, 1970) (Supplementary Table S3 and Figure 3).

### Organic Acids

**FIGURE 4:**
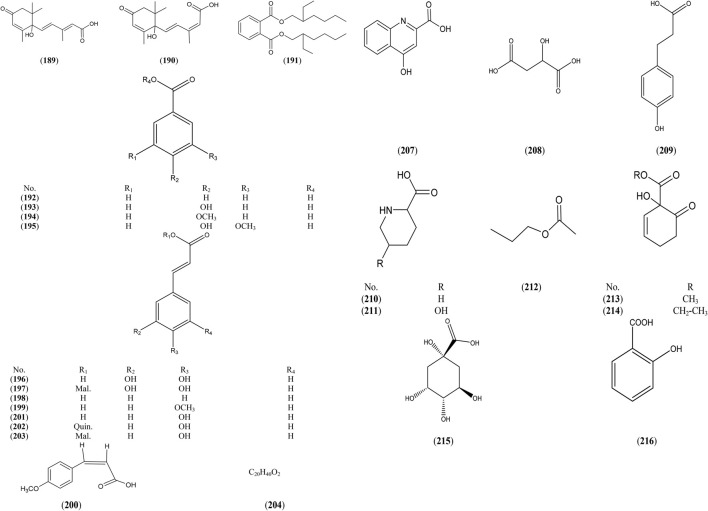
Structures of reported organic acids from genus *Salix.*

*Salix* species are rich sources for phenolic acids, either in free or esterified form, as benzyl, cinnamyl or phenyl ethyl esters. The aromatic acids are either benzoic or cinnamic acid derivatives: benzoic acid derivatives as *p*-hydroxybenzoic, *p*-anisic, gallic, salicylic, gentisic, vanillic, 2-amino-3-methoxy benzoic and protocatechuic acids, while hydroxycinnamic acid derivatives as *p*-coumaric, caffeic, isoferuolic, and feruolic acids, (Supplementary Table S4 and Figure 4).

The higest number of organic acids were detected in *S. purpurea* L., *S. alba* L. bark (Agnolet et al., 2012) which contain compounds (192–194, 198–200, 214), *S. tetrasperma* Roxb. flowers and bark (Sobeh et al., 2019; Mostafa et al., 2020) which contain compounds (197, 202, 203, 204, 205–206, 208, 209, 215).

### Simple Phenolics

**FIGURE 5:**
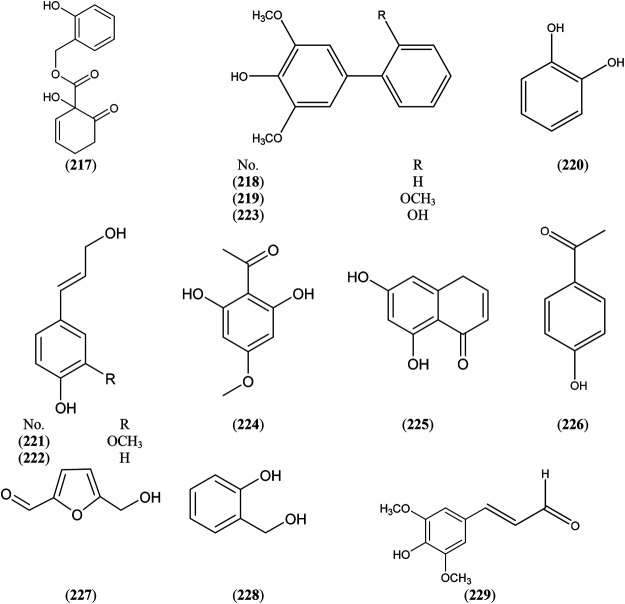
Structures of reported simple phenolics from genus *Salix.*

Genus *Salix* comprises a vast variety of simple phenolic compounds (Phenolic acids and their derivatives) (Tuberoso et al., 2011). *S. capensis* Thunb. bark (Masika et al., 2005), *S. acutifolia* Willd. bark (Zapesochnaya et al., 2002), *S. subserrata* Willd. bark (Hussain et al., 2011), *S. caprea* L. inflorescence (Ahmed et al., 2017) were characterized by the accumulation of salicyl alcohol 228) which is the basic nucleus for salicinoids. Also, *S. caprea* L. wood was characterized by the accumulation of different simple phenolics as aucuparin (218), methoxyaucuparin (219), coniferyl alcohol (221), *p*-coumaryl alcohol (222), 4,2′-dihydroxy-3,5-dimethoxybiphenyl 223) and sinapylaldehyde 229) (Malterud and Dugstad, 1985; Pohjamo et al., 2003), as illustrated in Supplementary Table S5 and Figure 5.

### Sterols and Terpenes

**FIGURE 6:**
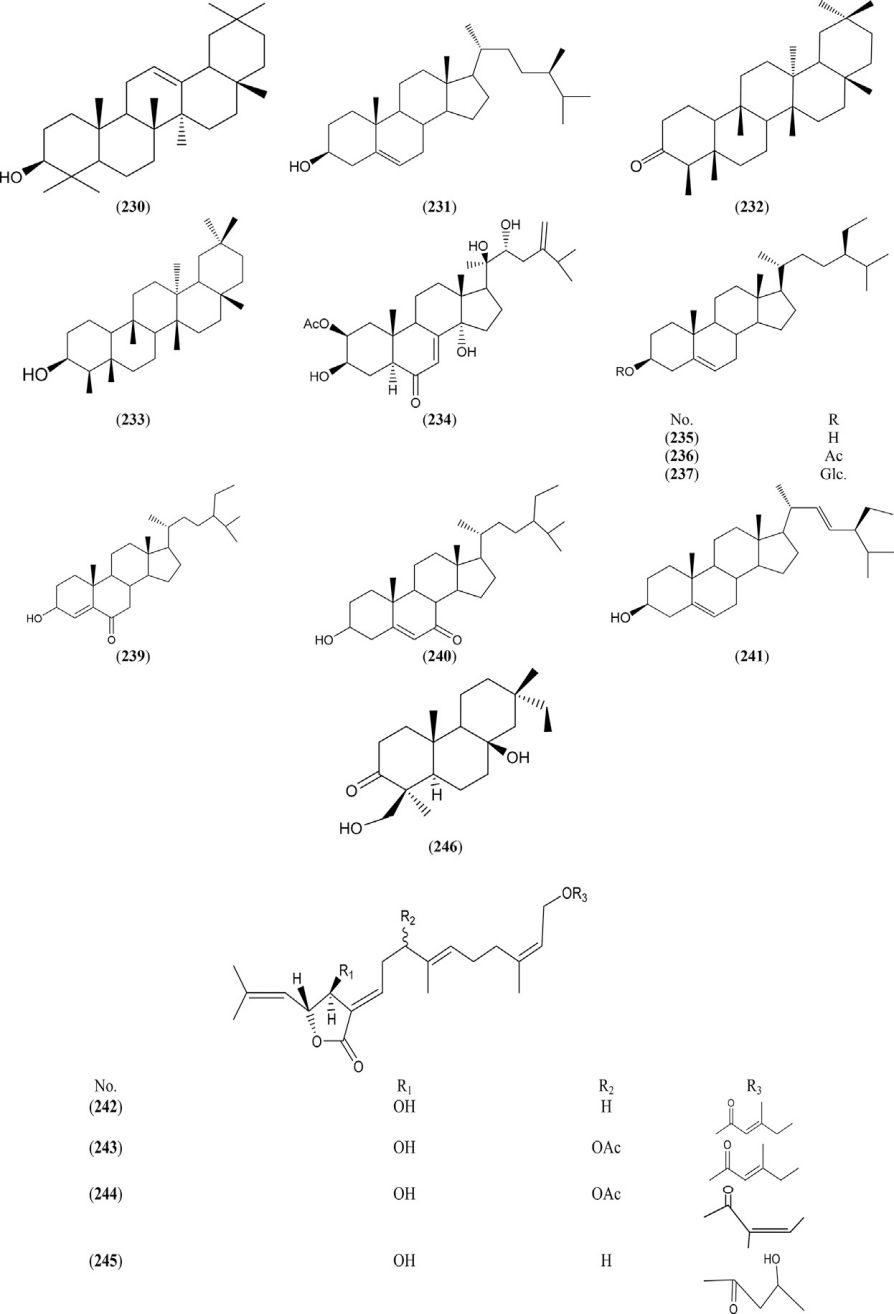
Structures of reported sterols and terpenes from genus *Salix.*

The highest number of sterols and triterpenes was detected in *S. cheilophila* C. K. Schneid. twigs (Shen et al., 2008), *S. tetrasperma* Roxb. bark, leaves and flowers (El-Shazly et al., 2012; Sobeh et al., 2019), *S. subserrata* Willd. leaves (Balbaa et al., 1979), *S. denticulate* erial parts (Rawat et al., 2009), *S. babylonica* L. roots (Singh et al., 2017), *S. subserrata* Willd. bark and leaves (Hussain et al., 2011). Whereas phytane and pimarane diterpene were found to be the major constituents in *S. cheilophila* C. K. Schneid. twigs (Shen et al., 2008), as illustrated in Supplementary Table S6 and Figure 6.

### Lignans

**FIGURE 7:**
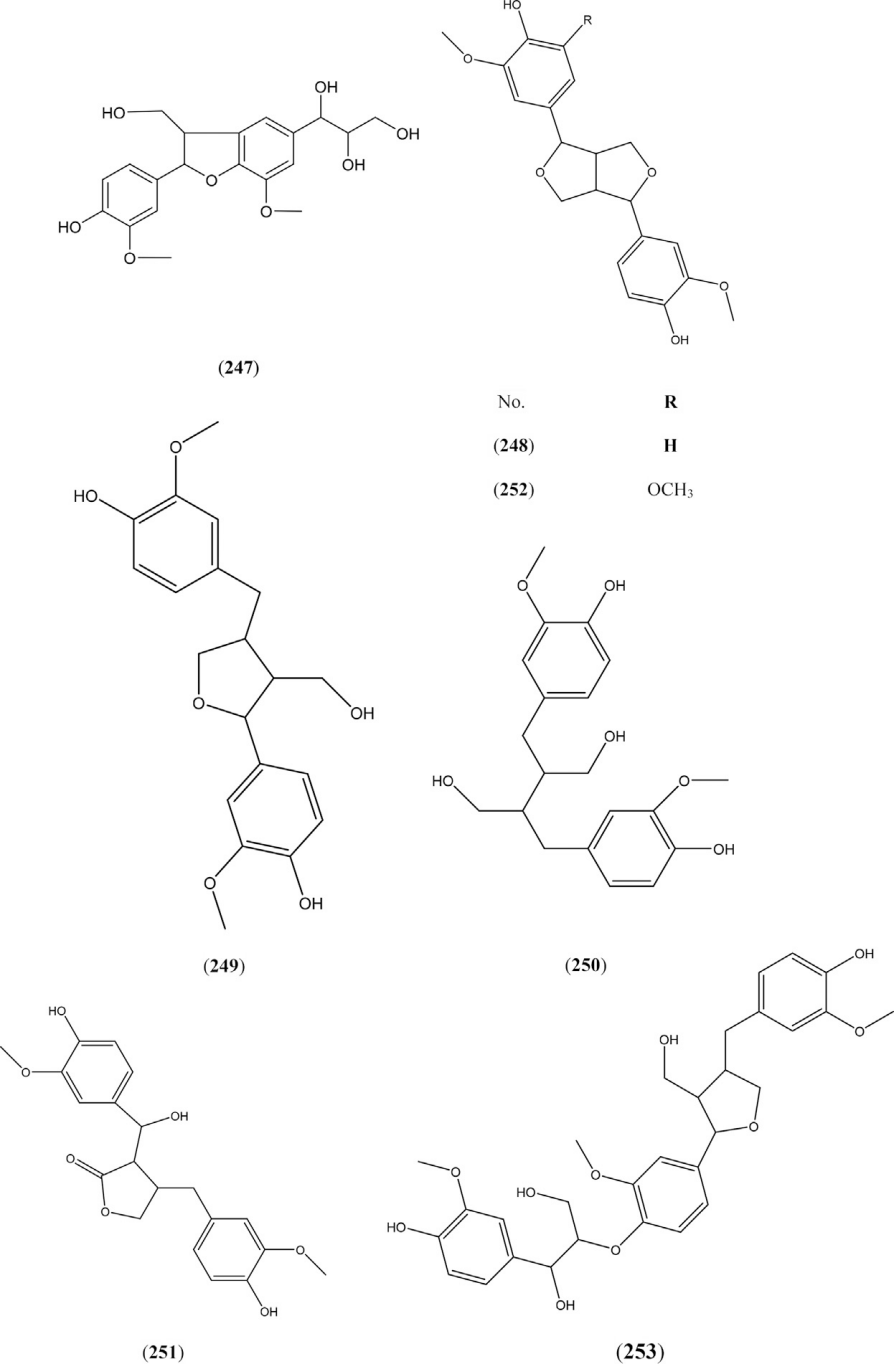
Structures of reported lignans from genus *Salix.*

Sisymbrifolin a lignan derivative 247) had been isolated from the bark of *S. alba* L. (Du et al., 2007)*.* Recently, pinoresinol (248), lariciresinol (249), secoisolariciresinol (250), 7-hydroxymatairesinol (251), medioresinol (252), and lariciresinol-sesquilignan 253) were detected in the biomass of five willow sp. cultivated in Quebec, Canada (Brereton et al., 2017) as illustrated in Figure 7.

### Volatiles

**FIGURE 8:**
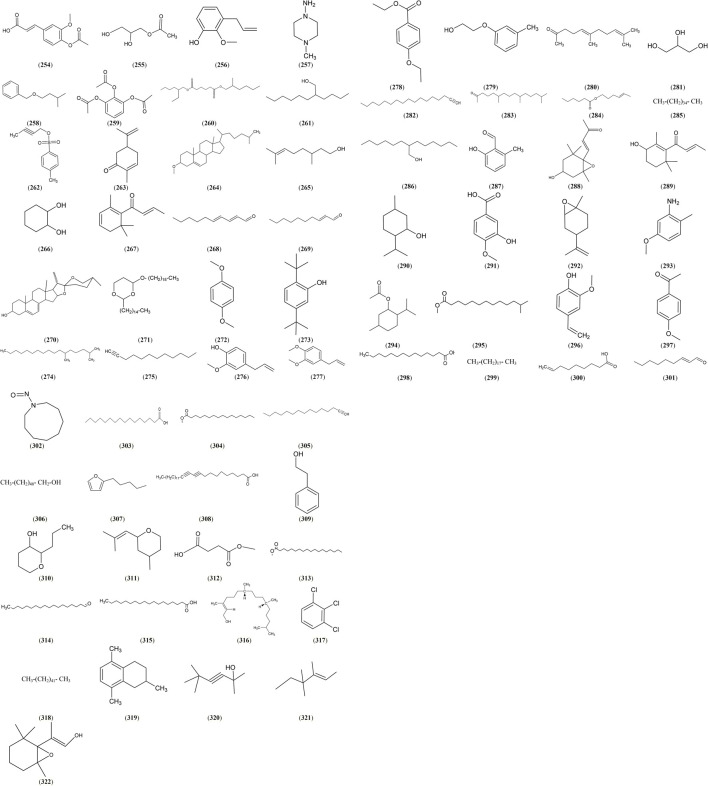
Structures of reported volatiles and fatty acids from genus *Salix.*

Terpenes (hemi-, mono- and sesqui-terpenes) and non-terpene (aliphatic, aromatic acids, their esters, carbonyl compounds and hydrocarbons) volatiles were identified in the genus *Salix*. The highest percent of volatiles and fatty acids was reported in *S. caprea* L. inflorescence (Ahmed et al., 2017), and the leaves of *S. egyptiaca* L. (Karimi et al., 2011), *S. babylonica* L. (Salem et al., 2011), and *S. alba* L. (Zarger et al., 2014) (Supplementary Table S7and Figure 8).

### Traditional Uses

*Salix* plants have been used medicinally since antiquity and have been linked to the discovery of acetylsalicylic acid and aspirin. These plants had been traditionally used to treat painful musculoskeletal joint pain conditions, inflammation, and fever. Salicin is a major pharmacologically active metabolite in *Salix* and hydrolyzes in the gastrointestinal tract to confer salicyl alcohol and d-glucose. The latter is oxidized, upon absorption, into salicylic acid, the active drug which inhibits cyclooxygenases (COX I, II) (Mahdi, 2010).

*S. egyptiaca* L (Musk Willow) was important in the Middle East, especially in Iran, as it has been traditionally used to treat anemia and vertigo, as a cardiotonic agent, and also in the preparation of local candies as a fragrance additive (Asgarpanah, 2012). *S. alba* L (white willow), had used in folk medicine to treat fever, chronic and acute inflammation, pain and infection (Zengion and Yarnell, 2011; Maistro et al., 2019). *S. tetrasperma* Roxb. had been used to treat diseases such as epilepsy, diabetes, fever, rheumatism, piles, swellings, stones in bladder, dysentery, wound, ear pain, cough and cold (Prashith Kekuda et al., 2017). *S. alba* L. bark is traditionally used for treatment of flu, rheumatism, fever and headache (Van Wyk and Wink, 2018).

### Pharmacological Activity

Different *Salix* species and the isolated compounds as salicylic acid and salicin have been utilized in folk medicine to treat rheumatic diseases, back pain, toothache, headache, and menstrual cramps (Highfield and Kemper, 1999). They exert analgesic, anti-inflammatory, antioxidant, anticancer, cytotoxic, antidiabetic, antimicrobial, anti-obesity, neuroprotective and hepatoprotective activities. The main targets of salicylic acid are cyclooxygenases (COX I, II) which are key enzymes of pathway to prostaglandins which control inflammation and pain. The available scientifically based reports on biological activities of genus *Salix* are summarized in Tables 1–8.

**TABLE 1:** Anti-bacterial activity of *Salix* species.

Bacteria	Extract/Compound	Used method	Effects	References
*B. subtilis S. aureus E. coli P. eruginosa*	*S. capensis* thunb. Bark catechol 2-hydroxybenzyl alcohol	Bioautographic assay on TLC plate, microplate dilution method broth culture	MIC = 62.5–250 µg/ml	Masika et al. (2005)
*S. mutans S. aureus Lactobacillus* sp	*S. alba* L. bark methanol extract	Disc diffusion method	MIC = 125 μg/ml 250 μg/ml for lactobacillus	Fayaz and Sivakumaar (2014)
*E. coli Salmonella enterica*	Aqueous extracts of *S. babylonica* L. leaves	Agar-gel diffusion method and twofold serial dilutions on mueller-hinton s agar	Inhibition zones with an average diameter of 13.38 ± 2.22 mm MIC50 = 70.4 ± 17.41 mg/ml	Popova and Kaleva (2015)
*E.coli, S.aureus Listeria monocytogenes*	Hydroalcoholic extract, fractions, and subfractions of *S. babylonica* L	Broth microdilution method	MIC = 0.78 mg/ml for *Listeria monocytogenes*, MIC = 0.39 mg/ml for *S. aureus*	González-Alamilla et al. (2019)
*P. eruginosa*	Methanol extracts of *S. tetrasperma* roxb. Stem bark and flower	Broth microdilution method, skim milk agar method	Inhibition of swimming and swarming motilities, and proteolytic and hemolytic activities	Mostafa et al. (2020)

**TABLE 2:** Antifungal, anthelmintic and anti-retroviral activity of *Salix* species.

Micro-organism	Extract/compounds	Used method	Effects	References
Fungi
*Fusarium oxysporum*	Ethanol extract of *S. babylonica* L. root	Poisoned food technique	Good fungicidal activity at 20% concentration	Sati et al. (2013)
*Candida guilliermondii*, *C. glabrata* and *C. parapsilosis*	Methanol extract of *S. alba* L. leaves	Broth microdilution method, filter disc assay and growth curve study	MIC = 800 μg/ml, 800 μg/ml and 1,600 μg/ml respectively. Inhibition i.e. 12 mm for *C. glabrata* followed by 11 mm measured in *C. parapsilosis*. *C. guilliermondii* inhibition was 10 mm	Zarger et al. (2014)
Parasites
*Hemonchus contortus, Eimeria Cooperia, Chabertia, Dictyocaulus, Moniezia,* and *Ostertagia*	Leaves of *S. babylonica* L. extract	Oocyst and egg count technique in goat and sheep	20 ml oral doses decrease oocyst and egg count in both species	Salem et al. (2017)
*Bonostomum* sp*., Strongiloides papillosus*, and *Nematodiruss pathiger Nematodirus battus*	Leaves extract of *S. babylonica* L	Salt floatation technique and McMaster method	The extract caused egg and worm count reductions in lamb feces by 47% vs. the control lambs	Hernandez et al. (2014)
Virus
HIV-1	*S. egyptiaca* L. Pl extract	XTT method. Inhibition of p24 Ag production level assay	The IC_50_ in HeLa infected cells was 45 μg/ml 100 μg/ml extract inhibited the production of HIV-1 p24 Ag by more than 80%	Eftekhari et al. (2014)

**TABLE 3:** *Invitro* antioxidant activity of *Salix* species.

Plant part	Extract/compound	Method	Effects	References
Stem and leaves	Four sulfated flavonoids (taxifolin-7-sulfate, dihydrokaempferol-7-sulfate, eridictyol-7-sulfate andnaringenin-7-sulfate) isolated from hybrid species of *Salix*×*alberti* L	DPPH	7-Sulfation of taxifolin and eriodictyol attenuated but does not remove antioxidant activity	Noleto-Dias et al. (2020)
Leaves	Methanol extracts of *S. purpurea* L.*, S. cinerea* L.*, Salix×smithiana* willd.*, S. alba* L.*, S. eriocephala* michx*., Salix×rubra* huds	DPPH	The scavenging effect ranged between 33.6 (*S. purpurea* L.) and 45.7% (*S. cinerea* L.), 50.7 (*S. purpurea* L.) to 56.3% (*Salix×rubra* huds.)	Gąsecka et al. (2017)
Leaves	Ethyl acetate extract of *S.tetrasperma* roxb	DPPH assay	IC_50_ = 65.89 μg/ml	Januarti et al. (2019)
Leaves	Methanol extract of *S. mucronata* andersson	DPPH, ABTS and TAC assays	DPPH (EC_50_ = 98.76 ± 0.46 (µg/ml), ABTS = 45.83 ± 0.32 mm, trolox eq./100 gm extract and TAC = 199.18 ± 2.19 mg equivalent of ascorbic acid/g extract). EtOAc fraction derived from MeOH (85%) extract demonstrated the highest antioxidant potential; DPPH EC_50_ = 50.19 ± 0.24 (µg/ml), ABTS = 76.22 ± 1.61 (mm trolox eq./100 gm extract) and TAC = 249.86 ± 3.74 (mg equivalent of ascorbic acid/g extract)	El-Sayed et al. (2015)
Male inflorescence	Methanol extract of *S. egyptiaca* L	DPPH and the folin–Ciocalteu method	Butanol fraction showed the highest antioxidant potential with an IC_50_ value of 27.7 µg/ml	Sonboli et al. (2010)
Flowers	Ethanol extract of *S. caprea* L	DPPH, superoxide hydrogen peroxide and nitric oxide scavenging assay	At a concentration of 250 μg/ml, 85.04% of DPPH radicals and at µg/mL 45.97%, 17.97% and 56.53% of O_2_·−, H_2_O_2_ and NO, respectively, were scavenged by the *S. caprea* L. flower extract	Alam et al. (2006)
Leaves, bark, catkins	Cyclohexane, butanol, ethanol and water extract of *S. egyptica* L	DPPH assay	Ethanol extract of the bark (highest activities, IC_50_ = 19 μg/ml)	Enayat and Banerjee (2009)
Bark	Hot ethanol extract of *S. alba* L	DPPH and folin-ciocalteu method	Free radical scavenging activity values ranged between 12.50, 37.50 and 80.00% of 10, 50 and 100 μg/ml, respectively	Sulaiman et al. (2013)
Bark	*S. alba* L., S. *daphnoides* Vill., *S. purpurea* L., and *S. daphnoides* Vill. x *purpurea* L. hybrid willow clones	ABTS	*S. daphnoides* Vill. x *purpurea* L. extracts were the most active ones.	Gawlik-Dziki et al. (2014)
Leaves and young stems	Hydroethanolic extract of *S. alba* L.	DPPH	IC_50_ =19.1 µg/ml.	Zabihi et al. (2018)
Leaves and male inflorescence catkin	*S. matsudana* Koidz. *S. aegyptiaca* L. *S. babylonica* L. *S. excelsa* S. G. Gmel. *S. acmophylla* Boiss.	DPPH, superoxide, nitric oxide and hydrogen peroxide radical scavenging activity	DPPH results ranged from 40.08% (*S. excelsa*) to 91.94% (*S. aegyptiaca* L.) and *S. excelsa* S. G. Gmel. displayed the potent superoxide (99.00%) and nitric oxide (71.73%) scavenging potential. Similar activities were found for hydrogen peroxide radical scavenging (50%) for *S. matsudana* Koidz.*, S. acmophylla* Boiss. and *S. babylonica* L.. Male inflorescence catkin extracts, *S. excelsa* S. G. Gmel (70.63%), *S. acmophylla* Boiss. (60.25%) and *S. matsudana* Koidz. (62.37%) presented the most activities in DPPH, nitric oxide and hydrogen peroxide, respectively. The *S. excelsa* S. G. Gmel*, S. aegyptiac*a L. and *S. babylonica* L. showed 99% superoxide radical inhibition.	Tavakoli et al. (2016)
Bark	Gallic acid, quercetin, rutin, vanillin and acetylsalicylic acid obtained from *S. aegyptiaca* L.	DPPH	gallic acid > quercetin >rutin> vanillin > acetylsalicylic acid.	Nauman et al. (2018)
Bark	Ethanol extract of *S. aegyptiaca* L.	DPPH	Ethyl acetate fraction showed the highest activity (11 ± 1 μg/ml).	Ceyhan (2014)
Bark	*S. alba* L.	DPPH	All granulometric classes revealed a high antioxidant activity. The best results were obtained for the 50–100 μm granulometric class.	Zaiter et al. (2016)
Flowers	Methanol extract of *S.tetrasperma* Roxb	TAC	30.97 ± 2.6, 26.8 ± 2.1 U/L for the extract and ascorbic acid, respectively.	Sobeh et al. (2019)
Bark	*S. atrocinerea* Brot.*, S. fragilis* L., *and S. viminalis* L. bark polar extracts	DPPH and ABTS.	Strong free radical scavenging activity (5.58–23.62 µg mL−1 IC_50_ range.	Ramos et al. (2019)
Leaves and bark	n- Hexane, dichloromethane, ethyl acetate and n- butanol extracts of *S. subserrata* Willd.	DPPH and FRAP assays.	IC_50_ µg/mL = 9.30 - 206.67 for DPPH assay and 2.90-26.89 mM FeSO_4_/mg extract for FRAP assay.	Tawfeek et al. (2019)
bark and leaves	*S. alba* L.*, S. amplexicaulis* Bory & Chaub.*, S. babylonica* L*.* *, S. eleagnos* Scop.*, S. fragilis* L.*, S. purpurea* L. *and S. triandra.* L.	DPPH and OH radical scavenging assay.	IC_50_ of DPPH ranged from 1.83–7.79 µg/mL in bark and 1.95–8.07 µg/mL in leaves extracts of different species of the genus *Salix*	Gligorić et al. (2019)

**TABLE 4:** *In vitro* antiproliferative effects of *Salix* species.

Extract/compound	Cell line	Methods	Results	Mechanism of action	References
Aqueous extract from *S. safsaf* forsk	AML	Trypan blue exclusion test	Killed most of the blasts of acute myeloid leukemia (AML, 73.8%)	Cells are killed through denaturation of some enzymes and proteins that are induced by salicin and saligenin	El-Shemy et al. (2003)
Aqueous extract of leaves extract of *S. safsaf* forsk. Salicin and saligenin	ALL and AML	Trypan blue exclusion test	A remarkable destruction of lymphoblasts (75%) was observed after 24 h incubation of the mononuclear ALL cells with extract. Similar trends were observed for mononuclear AML cells. The mean viability of willow extract treated cells was 26.2%	Unknown receptors on the surface of leukemic cells may be binding with *Salix* extract compounds and leading to DNA destruction	El-Shemy et al. (2007)
Salicylalcohol derivatives, flavonoids, proanthocyanidins, and salicin isolated from willow bark extract BNO 1455	Human colon cyclooxygenase-2 (COX-2)-positive HT 29 and (COX-2)-negative HCT 116 or lung COX-2 proficient a 549 and low COX-2 expressing SW2 cells	WST-1 assay and propidium iodide uptake by flow cytometry, annexin V adhesion using flow cytometry for apoptosis	GI_50_ 33.3–103.3 μg/ml for flavonoids and proanthocyanidins fractions and 50.0–243.0 μg/ml for salicyl alcohol derivatives and extract	ND	Hostanska et al. (2007)

**TABLE 5:** *In vivo* anticancer effects of *Salix* species.

Extract/compound	Doses	Route of administration	Methods	Effects	Mode of action	References
Aqueous extract from the young developing leaves of willow (*S.safsaf* forsk.)	0.2 and 0.6 ml of extract (10% w/v)	Oral	EACC were injected into the intraperitoneal cavity of mice	The willow extract reduced the tumor growth and delayed the death was delayed	Promote apoptosis, cause DNA damage, and affect cell membranes and/or denature proteins	El-Shemy et al. (2007)
Acetone soluble fraction of *S.caprea* L.flowers	0.5, 1.0 and 1.5 mg/kg	Topical application on the skin	7,12-Dimethyl benz [a] anthracene DMBA-initiated croton oil (phorbol ester)mice	Reductionin tumor incidence and number of tumors per mouse ranging from 20 to 50% and 50–63%	Intercept the free radicals and protect cellular macromolecules from oxidant damage. Effectiveness in inhibiting the ornithine decarboxylase activity and maintaining the activity of phase II enzymes after toxicant exposure	(Sultana and Saleem (2004)

**TABLE 6:** *In vivo* neuroprotective effects of *Salix* species and their major constituents.

Extract/Compound	Doses	Route	Model	Effect	References
Ethanol and aqueous extracts of *S. tetrasperma* roxb. Leaves	200 and 400 mg/kg	Oral	Mice	Decrease locomotor activity indicating CNS depressant activity in mice and has muscle relaxant activity	Virupaksha et al. (2016)
Methanol extract of *S. tetrasperma* roxb. FLowers	200 and 400 mg/kg	Oral	CCI rat model	Relieve hyperalgesia and allodynia responses	Sobeh et al. (2019)

**TABLE 7:** *In vivo* hepatoprotective effects of *Salix* species and their major constituents.

Extract/Compound	Doses	Route	Model	Effect	References
Ethanol extract of *S. subserrata* willd. Flowers	150 mg/kg	Oral	CCl_4_-induced chronic hepatotoxicity in rats	The elevated serum levels of intracellular liver enzymes and the expression levels of TNF-α and NFkB proteins were reduced	Wahid et al. (2016)
*S. caprea* L. flowers	50, 100, 150 mg/kg	Oral	Mice injected with ferric nitrilotriacetate (FeNTA)	Decreased hepatic lipid peroxidation, increased hepatic glutathione (GSH) content and the activities of antioxidant enzymes (catalase (CAT), glutathione reductase (GR) and glutathione peroxidase)	Alam et al. (2006)

**TABLE 8:** *In vivo* anti-obesity and anti-lipidemic effects of *Salix* species and their major constituents.

Extract/compound	Doses	Route of administration	Model	Effects	References
Ethanol extracts prepared from *S. babylonica* L. leaves	2.5 or 10 g (extract)/kg food	Supplemented in diet	HFD mice	Decreased body weight and parametrial adipose tissue weight	Liu (2012)
Ethanol extracts prepared from *S. babylonica* L. leaves	10%	Supplemented in diet	Rats orally administered 1 ml of a lipid emulsion composed	The extracts inhibited the elevation of blood triacylglycerol	Liu (2012)
Polyphenol fractions of *S. matsudana* koidz. Leaves	5%	Supplemented in diet	HFD mice	Decreased body weight and reduced the hepatic total cholesterol content	Han et al. (2003a)

### Antimicrobial Effects of Salix

Multidrug-resistant bacteria are widely spread, and natural resources have been used as a means of discovering novel antibacterial compounds as they offer limitless opportunities for the discovery of new agents, particularly against multidrug resistant bacteria.

The main methods used to evaluate the antimicrobial activity of *Salix* extracts are disc diffusion assays, agar well diffusion, broth microdilution methods and the assessment of antibiofilm function (Masika et al., 2005; Fayaz and Sivakumaar, 2014; Popova and Kaleva, 2015; Mostafa et al., 2020). As detailed in Table 1, microbial growth inhibition zones and percentages along with minimum inhibitory concentrations (MICs) displayed the potential of *Salix* species as substantial antimicrobials and predict their efficacy as functional foods (Mostafa et al., 2020).

### Antibacterial Activity

Many previous studies evaluated the antibacterial activity of *Salix* plants and active constituents of their extracts against different types of bacteria such as *Pseudomonas eruginosa*, *Escherichia coli, Staphylococcus aureus* and *Bacillus subtilis*, dental biofilm forming bacteria (*Streptococcus mutans* and *Lactobacillus*), and *Salmonella enterica* (Table 1). Catechol and 2-hydroxybenzyl alcohol derived from the bark of *S. capensis* Thunb. were previously tested for their antibacterial activity. Both compounds exhibited similar antibacterial activity against *P. eruginosa* (Masika et al., 2005). Moreover, *Salix alba* L. bark extract demonstrated antimicrobial activity against the dental biofilm forming bacteria with MIC of 125 μg/ml. Furthermore, it also exhibited a moderate potential against the *Staphylococcus aureus* but the least activity was observed against *E. coli* (Fayaz and Sivakumaar, 2014). Previous studies also showed that the twigs aqueous extract with leaves of *S. babylonica* L. exhibited potent antimicrobial properties against Gram-negative bacteria (*E. coli*, *Salmonella enterica*, MIC_50_ is 70.4 ± 17.41 mg/ml) with a comparable activities to thiamphenicol (The broad spectrum antibiotic). Its effects cover Gram-positive bacteria such as *S. aureus* (Popova and Kaleva, 2015). A recent study performed in our laboratories tested the extracts of both stem bark and flowers of *S. tetrasperma* Roxb. for anti-quorum sensing activity against *Pseudomonas eruginosa*. Both extracts inhibited *P. eruginosa* bacterial growth at 40 mg/ml; however, the bacterial viability was not affected by 1/4 and 1/8 MIC concentrations. When the extracts were tested as anti-quorum sensing agents, they impaired virulence of *P. eruginosa* by declining its swimming and swarming motilities and reducing its hemolytic and proteolytic properties (Mostafa et al., 2020).

### Antifungal Activity

Poisoned food technique, broth microdilution method, filter disc assay and growth curve study methods were used to determine the antifungal properties of *Salix* extracts (Table 2). The antifungal activity was evaluated against *Candida guilliermondii*, *C. glabrata*, *C. parapsilosis* and *Fusarium oxysporum*.

### Anthelmintic Activity

The anthelmintic potential of *Salix* species to inhibit gastrointestinal and pulmonary parasites in animals was studied. The anthelmintic activity was evaluated against *Ostertagia*, *Moniezia*, *Dictyocaulus, Eimeria*, *Chabertia, Cooperia*, and *Hemonchus contortus* (Table 2). It was reported *Salix babylonica* L (at dose of 20 ml, weekly) was effective against the main parasite species detected in sheep (*Eimeria*spp., *Dictyocaulus* spp., and *Chabertia* spp.) more than the most common parasites in goats in southern Mexico farms (*Dictyocaulus* spp. and *Chabertia* spp.) (Salem et al., 2017).

### Anti -HIV Activity

Human immunodeficiency virus (HIV) infection that causes acquired immunodeficiency syndrome (AIDs) represents a major health problem worldwide. Chemical anti-retroviral agents are usually used to treat AIDs patients. However, they possess many adverse effects and resistance emerged for many of them. Recently, novel anti-retroviral agents isolated from medicinal plants, played an essential role to replace synthetic drugs. One study investigated the anti-retroviral effects of *S. egyptiaca* L. extract. Results of this study and bioinformatics analyses suggested that the plant had anti-HIV properties and might be a substantial candidate for AIDS patients (Table 2) (Eftekhari et al., 2014).

### Antioxidant Activity

Reactive oxygen species (ROS) are associated with several human diseases, such as inflammation, diabetes, ulcers, autoimmune and cardiovascular diseases, viral infections and cancer (Howlett, 2008; Rubió et al., 2013; Salem et al., 2020). Most of the activities of *Salix* species were attributed to the presence of several polyphenolic with robust antioxidant activities (Table 3). The antioxidant effects of *Salix* extracts and their flavonoids were mainly assessed by DPPH, ABTS, FRAP, total antioxidant capacity (TAC) assays, Folin-Ciocalteu method, β-carotene bleaching, lipid peroxidation capacity, inhibition of linoleic acid oxidation, superoxide anion radical scavenging, and alkyl radical scavenging assays (Ceyhan, 2014; Gawlik‐Dziki et al., 2014; Tavakoli et al., 2016; Zaiter et al., 2016; Nauman et al., 2018; Zabihi et al., 2018; Gligoric’ et al., 2019). A recent study from our lab investigated the possible effect of *S. tetrasperma* Roxb. extract on neuropathic pain and its mechanism of action showed a potent *in vitro* and *in vivo* antioxidant effects (Sobeh et al., 2019). Furthermore, *S. atrocinerea* Brot.*, S. fragilis* L. and *S. viminalis* L. showed antioxidant effects mediated by their polyphenolic contents (Ramos et al., 2019). Another study from our laboratory showed that *S. subserrata* Willd. leaf extracts contained isorhamnetin-3-*O*-*β*-d-rutinoside, triandrin, gallocatechin, tremuloidin, aromadendrin, salicin, and chrysoeriol-7-*O*-glucuronid and exerted antioxidant effects against oxidative stress in *Caenorhabditis elegans* (Tawfeek et al., 2019).

### Anti-Inflammatory Activity

Inflammation is a frequent condition because of exposure to different stimuli including microbial infection and wounding. It decreases the spread of infection, followed by resolution and the restoration of normal structural and functional of affected tissues (Nathan and Ding, 2010). However, non-resolving inflammation contributes significantly to the pathogenesis of many diseases such as atherosclerosis, obesity, cancer, and inflammatory bowel disease. *Salix* extracts exert potent anti-inflammatory effects that are responsible for many biological effects. The hydroalcoholic extract of *S. tetrasperma* Roxb. in two dose levels (100 and 200 mg/kg) demonstrated anti-inflammatory effects in carrageenan induced rat paw edema model (Kishore et al., 2014). We showed previously that the flower extract of *S. tetrasperma* Roxb. has analgesic, antipyretic, and anti-inflammatory effects against carrageenan induced vascular permeability and carrageenan induced hind paw edema. It inhibited COX-1, COX-2 and LOX and suppressed elevated levels of TNF-a and NF-κB in chronic neuropathic pain model (Sobeh et al., 2019). Oral administration of *S. canariensis* extract significantly decreased writhing, moderately reduced formalin-induced pain and showed a promising dose-dependent anti-inflammatory activities. These effects were attributed to the presence of pentacyclic triterpenes and polyphenolics (Gutiérrez et al., 2017). An early study showed that *S. caprea* L. is a potent cyclooxygenase inhibitor (Tunon et al., 1995). Another study showed that S. *subserrata* Willd. and *S. tetrasperma* Roxb. showed anti-inflammatory effects against carrageenan induced hind paw edema due to the presence of phenolic glycosides mainly salicin as well as the flavonoids luteolin, quercetin and rutin (Karawya et al., 2010). *S. matsudana* Koidz. leaves methanol extract also showed significant inhibitory activities against cyclooxygenases (COX-1 and COX-2) due to the presence of matsudone, luteolin 7-O-glucoside and 4′,7-dihydroxyflavone (Li et al., 2008).

### Anticancer Activity

There are several risk factors that can increase the development of cancer that have a basis of low-grade inflammation and oxidative stress. Therefore, targeting inflammatory pathways and suppressing oxidative stress may contribute to inhibition of initiation, proliferation and even cancer metastasis and subside resistance to chemotherapy and radiation. *Salix* extracts, by possessing both anti-inflammatory and potent antioxidant potential, are promising natural sources in fighting cancer. The antiproliferative activities of *Salix* extracts were determined by cell viability percentages and IC_50_ values using several *in vitro* assays. The most commonly utilized cancer cell lines were human acute lymphoblastic leukemia (ALL cells), human acute myeloid leukemia cells (AML cells), PC3 cells (Prostate cancer cells), Hep G2 cells (Liver cancer cells), HCT116 (Colorectal cancer cells), MCF7 (Breast cancer cells), HT-29 and HCT 116 (human colon COX-2 positive and negative cells respectively), A549, SW2 cells, and human lung cancer cell line (H1299).

It was observed that a fraction of *Salix* extracted by non-polar solvents such as (petroleum ether, ether, and chloroform) has the minimum killing potential against AML cells while fraction extracted by polar solvents such as 70% ethanol and water has major destructive effect on AML cells (El-Shemy et al., 2003). Thus, *Salix* cytotoxic activity could be attributed to the polyphenolics, tannins, and glycosides, that are commonly dissolved in water or ethanol solutions including salicin and saligenin. When salicin is tested against leukemic cells it caused destruction of myeloblasts by 70–75%. Eight compounds isolated from *S. hulteni* Flod (1-p-coumaroyl-β-D-glucoside, aromadendrin, catechin, 4-hydroxyacetophenone, picein, sachaliside 1, naringenin and dihydromyricetin) were tested for their cytotoxic potential against brine shrimp and a human lung cancer cell line (H1299). Naringenin, aromadendrin, catechin, and 1-*p*-coumaroyl-β-D-glucoside showed mild cytotoxic activity, with dihydromyricetin showing the strongest cytotoxic effects. 4-Hydroxyacetophenone, picein, and sachaliside one did not show a significant cytotoxic activity indicating that flavonoid compounds are responsible for the cytotoxic effects of *S. hulteni* Flod. (Jeon et al., 2008). Brine shrimp lethality test is commonly used to test cytotoxic effects of natural products. The methanol extract of *S. nigra* exerted concentration dependent cytotoxic effects against brine shrimp indicating promising cytotoxic effects (Ahmed et al., 2016). Willow bark extract (A pharmaceutically used extract BNO 1455) and its fractions (flavonoids, proanthocyanidins, salicyl alcohol derivatives) showed dose dependent cytotoxic effects against human colon and lung cancer irrespective of their COX-2 selectivity (Hostanska et al., 2007). *S. caprea* L. exerted a protective effect against phorbol ester induced skin tumor promotion when applied to the skin of mice prior to the application of phorbol ester. Anti-tumor activity of *S. caprea* L. may be attributed to potent antioxidants constituents of *S. caprea* L. such as luteolin, dihydrokaempferol and quercetin (Sultana and Saleem, 2004).

### Neuroprotective Effect

Only few studies investigated the effect of *Salix* species on central and peripheral nervous system. Virupaksha et al. (2016) investigated the effects of *S. tetrasperma* Roxb. leaf extract on locomotor activity and muscle relaxant activity. They demonstrated that the extract decreased locomotor activity indicating central nervous system (CNS) depressant activity and induced a decrease in fall off time due to loss of muscle grip implying skeletal relaxation (Virupaksha et al., 2016). The CNS depressant activity of the extract was attributed to binding of flavonoids to gamma-aminobutyric acid (GABA) receptors in the CNS (Hossain et al., 2009). Another study from our laboratory investigated the possible protective effect of *S. tetrasperma* Roxb. on neuropathic pain model, chronic constriction injury of sciatic nerve model. In this work, we explored the effects of the extract on central and peripheral nervous system in this model. We showed that the extract improved hyperalgesia and allodynia, the major signs of neuropathic pain through inhibition of oxidative stress and inflammation in sciatic nerve and brain stem (Sobeh et al., 2019).

### Hepatoprotective Effects

*S. subserrata* Willd. flower extract showed marked hepatoprotective effects mostly through lowering the elevated liver enzymes and decreasing the protein levels of two inflammatory biomarkers (NF-κB and TNF-α) in carbon tetrachloride (CCl_4_)-induced liver damage model (Wahid et al., 2016). It also presented a remarkable ability to reduce lipid peroxidation and had antioxidant effects related to several active ingredients that include flavonoids such as quercetrin, luteolin-7-glucoside, rutin, and quercetin and phenolic compounds such as salignin and catechins.

### Anti-Obesity and Anti-lipidemic Effects

As shown in Table 8, remarkable anti-obesity and anti-lipidemic effects have been attributed to *Salix* extracts. The reduction of parametrial adipose tissue weight and body weight gain, the reduction of liver total cholesterol contents and inhibition of the elevated blood triacylglycerol are among the most prominent, directly attributed to its ability to inhibition of intestinal absorption of dietary fat (Liu, 2012). These effects have been mostly attributed to polyphenol fractions (apigenin-7-*O*-D-glucoside, luteolin-*O*-D- glucoside and chrysoeriol-7-*O*-D-glucoside) which inhibited palmitic acid incorporation into small intestinal brush border membrane vesicles (Han et al., 2003). It was reported that methanol extract of *S. pseudo-lasiogyne* H. Lév. twigs and salicortin derivatives reduced lipid accumulation in a concentration-dependent manner. They inhibited the differentiation of adipocytes in 3T3-L1 cells. The 2′,6′-*O*-acetylsalicortin exhibited the most potent inhibitory activity with IC_50_ = 11.6 μM. It remarkably downregulated the expressions of sterol regulatory element binding protein 1 (SREBP1c) and CCAAT/enhancer binding protein α (C/EBPα). Thus, salicortin derivatives possessed anti-adipogenic effects via down-regulation of SREBP1c and C/EBPα dependent pathways (Lee et al., 2013).

## Conclusion and Future Perspectives

The current review outlined the complete research progress in the phytochemistry, traditional use and pharmacology of genus *Salix* plant extracts and constituents. *Salix* extracts and some of its components exerted potent antioxidant, anti-inflammatory, antiproliferative, and antimicrobial properties confirming the traditional use of willow extracts in folk medicine. They also demonstrated substantial abilities in suppressing inflammatory pathways, both in cancer prevention and treatment, and in other chronic diseases. Thus, as a potential perspective, *Salix* extracts alone or their isolated active components should be examined more thoroughly, and its anti-HIV, hepatoprotective and neuroprotective therapeutic approach should also be discussed.

## Glossary

ABTS: 2,2′-azinobis-(3- ethylbenzothiazoline-6-sulfonic acid)
AIDs: Acquired immunodeficiency syndrome
Apif: Apiofuranosyl
Araf: Arabinofuranosyl
Arap: Arabinopyranosyl
CCl_4_: Carbon tetrachloride
C/EBPα: CCAAT/enhancer binding protein α
DPPH: 2,2-Diphenyl, one- Picryl Hydrazyl
EACC: Ehrlich ascites carcinoma cells
EtOAc: Ethyl acetate
FRAP: Ferric reducing antioxidant power
Glac: Galactosyl
Glc: Glucosyl
Gluc: Glucuronoyl
Hex: Hexosyl
HFD: High-fat diet
HIV: Human immunodeficiency virus
IC_50_: Half maximal inhibitory concentration
MeOH: Methanol
MIC: Minimal inhibitory concentration
NF-κB: Nuclear factor kappa-B
ORAC: Oxygen radical absorbance capacity
Pent: Pentosyl
Ph: Phenyl
Rh: Rhamanosyl
Rut: Rutinosyl
SREBP1c: Sterol regulatory element binding protein 1
TAC: Total antioxidant capacity
TFC: Total flavonoid content
TLC: Thin layer chromatography
TNF-α: Tumor necrosis factor-alpha
TPC: Total phenolic content
XTT: 2,3-bis-(2-methoxy-4-nitro-5-sulfophenyl)-2H- tetrazolium-5-carboxanilide
Xylp: Xylopyranosyl.
