# Supplementary material for: Phytochemistry, Pharmacology and Medicinal Uses of Plants of the Genus Salix: An Updated Review
Source: Front Pharmacol. 2021 Feb 12;12:593856. doi: 10.3389/fphar.2021.593856 (PMC7908037; doi:10.3389/fphar.2021.593856)
Supplement: Supplementary file 1 [file datasheet1.docx]

Supplementary Material

**Phytochemistry, Pharmacology and Medicinal Uses of Plants of the Genus *Salix*: An Updated Review**

Nora Tawfeek^1,2^, Mona F. Mahmoud^3^, Dalia I Hamdan^4^, Mansour Sobeh^1,5^, Nawaal Farrag^2^, Michael Wink ^1,^*, and Assem M. El-Shazly^2,^*

*Correspondence: wink@uni-heidelberg.de (M.W.); assemels2002@yahoo.co.uk (A.M.E.); Tel: +49 6221 54 4880 (M.W.), Fax: +49 6221 54 4884 (M.W.)

| **Supplementary TABLE S1.** Reported flavonoids from the genus *Salix*. | | | |
| --- | --- | --- | --- |
| **Compound** | | **Species** | **References** |
| **Name** | **No.** |  |  |
| 1. **Flavones** | | | |
| Apigenin | (**1**) | *S. acutifolia* Willd. | ([Shelyuto and Bondarenko, 1985](#_ENREF_96)) |
| Apigenin-7-*O*-D-glucoside | (**2**) | *S. acutifolia* Willd. | ([Shelyuto and Bondarenko, 1985](#_ENREF_96)) |
|  |  | *S. gilgiana* Seemen. | ([Mizuno et al., 1987](#_ENREF_69)) |
|  |  | *S. matsudana* Koidz. | ([Han et al., 2003b](#_ENREF_33)) |
| Apigenin-coumaroyl glucoside (terniflorin) | (**3**) | *S. tetrasperma* Roxb. | ([Sobeh et al., 2019](#_ENREF_99); [Mostafa et al., 2020](#_ENREF_71)) |
| Apigenin-7-*O*-galactoside | (**4**) | *S. babylonica* L. | ([Khatoon et al., 1988](#_ENREF_47); [Singh et al., 2017](#_ENREF_98)) |
| Apigenin-3ˋ-oxyethyl-7-*O*-glucoside | (**5**) | *S. matsudana* Koidz. | ([Li et al., 2013](#_ENREF_60)) |
| Chrysoeriol | (**6**) | *S. babylonica* L. | ([Liu et al., 2008](#_ENREF_63)) |
| Chrysoeriol-7-*O*-D-glucoside | (**7**) | *S. matsudana* Koidz. | ([Han et al., 2003b](#_ENREF_33)) |
| Chrysoeriol-7-*O*-glucuronide | (**8**) | *S. subserrata* Willd. | ([Tawfeek et al., 2019](#_ENREF_104)) |
| 4ˋ,7-Dihydroxyflavone | (**9**) | *S. matsudana* Koidz. | ([Li et al., 2008](#_ENREF_61)) |
| 5,7-Dihydroxy-3'-methoxyflavone | (**10**) | *S. tetrasperma* Roxb. | ([Januarti et al., 2019](#_ENREF_42)) |
| 2ˋ,5-Dihydroxy-4ˋ-methoxyflavone-7-*O*-*β*-D-glucopyranoside | (**11**) | *S. denticulata* Andersson | ([Rawat et al., 2009](#_ENREF_87)) |
| 7,3ˋ-Dihydroxy-4ˋ-methoxyflavone-5-*O*-*ß*-D-glucopyranosyl (6ˋˋ→1ˋˋˋ)-*ß*-D-glucopyranoside | (**12**) | *S. denticulata* Andersson | ([Semwal et al., 2011](#_ENREF_93)) |
| 5,3ˋ-Dihydroxy-4ˋ-*O*-angeloxyflavone-7-*O*-ß-D-glucopyranoside | (**13**) | *S. cheilophila* C.K.Schneid. | ([Shen et al., 2008](#_ENREF_97)) |
| Diosmetin | (**14**) | *S. denticulata* Andersson | ([Semwal et al., 2011](#_ENREF_93)) |
| Luteolin | (**15**) | *S. denticulata* Andersson | ([Semwal et al., 2011](#_ENREF_93)) |
|  |  | *S. matsudana* Koidz. | ([Li et al., 2008](#_ENREF_61)) |
|  |  | *S. acutifolia* Willd. | ([Shelyuto and Bondarenko, 1985](#_ENREF_96)) |
|  |  | *S. gilgiana* Seemen. | ([Mizuno et al., 1987](#_ENREF_69)) |
|  |  | *S. babylonica* L. | ([Zeid, 2006](#_ENREF_118); [Liu et al., 2008](#_ENREF_63); [González-Alamilla et al., 2019](#_ENREF_30)) |
| Luteolin-6-*C*-*β*-D-glucopyranoside (iso-orientin) | (**16**) | *S. babylonica* L. | ([Zeid, 2006](#_ENREF_118)) |
| Luteolin-5-*O*-ß-D-glucopyranoside | (**17**) | *S. cheilophila* C.K.Schneid. | ([Shen et al., 2008](#_ENREF_97)) |
| Luteolin-4ˋ-*O*-glucoside | (**18**) | *S. babylonica* L. | ([Khatoon et al., 1988](#_ENREF_47)) |
| Luteolin-7-*O*-*ß*-D-glucopyranoside (cynaroside) | (**19**) | *S. cheilophila* C.K.Schneid. | ([Shen et al., 2008](#_ENREF_97)) |
|  |  | *S. matsudana* Koidz. | ([Li et al., 2008](#_ENREF_61)) |
|  |  | *S. acutifolia* Willd. | ([Shelyuto and Bondarenko, 1985](#_ENREF_96)) |
|  |  | *S. caprea* L. | ([Nasudari et al., 1972](#_ENREF_73)) |
|  |  | *S. gilgiana* Seemen. | ([Mizuno et al., 1987](#_ENREF_69)) |
|  |  | *S. matsudana* Koidz. | ([Han et al., 2003b](#_ENREF_33)) |
|  |  | *S. babylonica* L. | ([Liu et al., 2008](#_ENREF_63); [González-Alamilla et al., 2019](#_ENREF_30)) |
|  |  | *S. subserrata* Willd. | ([Balbaa et al., 1979](#_ENREF_8)) |
| Luteolin-7-*O*-(6ˋˋ-*trans*-cinnamoyl)-*ß*-D-glucopyranoside | (**20**) | *S. gilgiana* Seemen. | ([Mizuno et al., 1987](#_ENREF_69)) |
| Luteolin-7-*O*-(6ˋˋ-feruloy1)-*ß*-D-glucopyranoside | (**21**) |  |  |
| Luteolin-7-O-(6ˋˋ-acetyl)-*ß*-D-glucopyranoside | (**22**) |  |  |
| Luteolin-7-*O*-(6ˋˋ-*p*-coumaroyl)-*ß*-D-glucopyranoside | (**23**) |  |  |
| Luteolin-7-*O*-*β*-D-glucopyranosyl-(1→6)-glucopyranoside | (**24**) | *S. denticulata* Andersson | ([Rawat et al., 2009](#_ENREF_87)) |
| 7-Methoxyflavone | (**25**) | *S. matsudana* Koidz. | ([Li et al., 2008](#_ENREF_61)) |
| 1. **Flavonols** | | | |
| Isorhamnetin-3-*O*-glucoside | (**26**) | *S. chaenomeloides*  Kimura | ([Mizuno et al., 1991](#_ENREF_70)) |
|  |  | *S. tetrasperma* Roxb. | ([Mostafa et al., 2020](#_ENREF_71)) |
| Isorhamnetin-3-*O*-rutinoside | (**27**) | *S. chaenomeloides*  Kimura | ([Mizuno et al., 1991](#_ENREF_70)) |
| Isorhamentinpentosyl-rutinoside | (**28**) | *S. tetrasperma* Roxb. | ([Sobeh et al., 2019](#_ENREF_99)) |
| Isorhamentin 3-*O*-rhamnoside | (**29**) |  |  |
| Isorhamentinpentosylhexoside | (**30**) |  |  |
| Kaempferide 3-O-hexoside | (**31**) |  |  |
| Kaempferol | (**32**) | *S. bordensis* Turcz. | ([Zhao et al., 2014](#_ENREF_120)) |
| Kaempferol-7,4ˋ-dimethyl | (**33**) | *S. bordensis* Turcz. | ([Zhao et al., 2014](#_ENREF_120)) |
| Kaempferol-7-*O*-glucoside | (**34**) | *S. babylonica* L. | ([Khatoon et al., 1988](#_ENREF_47); [Singh et al., 2017](#_ENREF_98)) |
| Matsudone A | (**35**) | *S. matsudana* Koidz. | ([Li et al., 2008](#_ENREF_61)) |
| Myricetin-3-*O*-β-D-glucoside | (**36**) | *S. subserrata* Willd. | ([Tawfeek et al., 2019](#_ENREF_104)) |
| Quercetin | (**37**) | *S. acutifolia* Willd. | ([Shelyuto and Bondarenko, 1985](#_ENREF_96)) |
|  |  | *S. bordensis* Turcz. | ([Zhao et al., 2014](#_ENREF_120)) |
|  |  | *S. subserrata* Willd. | ([Balbaa et al., 1979](#_ENREF_8)) |
| Quercetin-3-*O*-*β*-D-glucoside | (**38**) | *S. denticulata* Andersson | ([Semwal et al., 2011](#_ENREF_93)) |
|  |  | *S. acutifolia* Willd. | ([Shelyuto and Bondarenko, 1985](#_ENREF_96)) |
|  |  | *S. subserrata* Willd. | ([Balbaa et al., 1979](#_ENREF_8)) |
|  |  | *S. matsudana* Koidz. | ([Li et al., 2008](#_ENREF_61)) |
| Quercetin-3-*O*-rhamnoside | (**39**) | *S. chaenomeloides*  Kimura | ([Mizuno et al., 1991](#_ENREF_70)) |
| Quercetin-7-*O*-glucoside | (**40**) | S. *acutifolia* Willd. | ([Shelyuto and Bondarenko, 1985](#_ENREF_96)) |
|  |  | *S. pentandroides* A.K.Skvortsov | ([Kompantsev and Shinkarenko, 1973](#_ENREF_53)) |
| Quercetin-3-*O*-rutinoside (rutin) | (**41**) | *S. chaenomeloides*  Kimura | ([Mizuno et al., 1991](#_ENREF_70)) |
|  |  | *S. acutifolia* Willd. | ([Shelyuto and Bondarenko, 1985](#_ENREF_96)) |
|  |  | *S. subserrata* Willd. | ([Balbaa et al., 1979](#_ENREF_8)) |
| Quercetin-3-*O*-galactoside | (**42**) | *S. chaenomeloides*  Kimura | ([Mizuno et al., 1991](#_ENREF_70)) |
|  |  | *S. pentandroides* A.K.Skvortsov | ([Kompantsev and Shinkarenko, 1973](#_ENREF_53)) |
|  |  | *S. atrocinerea* Brot.*, S. fragilis* L. and *S.viminalis* L. | ([Ramos et al., 2019](#_ENREF_86)) |
| Quercetin-3-*O*-*β*-D-glucopyranuronide | (**43**) | *S. bordensis* Turcz. | ([Zhao et al., 2014](#_ENREF_120)) |
| Quercetin dihexoside | (**44**) | *S. tetrasperma* Roxb. | ([Sobeh et al., 2019](#_ENREF_99)) |
| Quercetin pentosyl-rutinoside | (**45**) |  |  |
| Quercetin pentosyl-hexoside | (**46**) |  |  |
| Rhamnetin | (**47**) | *S. bordensis* Turcz. | ([Zhao et al., 2014](#_ENREF_120)) |
| 1. **Flavanones** | | | |
| Eriodictyol | (**48**) | Willow bark extract (STW 33-I) | ([Freischmidt et al., 2010](#_ENREF_26)) |
|  |  | *S. alba* L. | ([Du et al., 2004](#_ENREF_16)) |
|  |  | *S.atrocinerea Brot., S. fragilis* L. and *S. viminalis* L. | ([Ramos et al., 2019](#_ENREF_86)) |
| Eriodictyol-7-sulfate | (**49**) | *S. integra* [Thunb.](https://en.wikipedia.org/wiki/Carl_Peter_Thunberg) *× S.suchowensis* W.C.Cheng ex G.Zhu | ([Noleto-Dias et al., 2020](#_ENREF_77)) |
| Eriodictyol-7-glucoside | (**50**) | Willow bark extract (STW 33-I) | ([Freischmidt et al., 2010](#_ENREF_26)) |
| Naringenin | (**51**) | *S. daphnoides* [Vill.](https://en.wikipedia.org/wiki/Vill.) | ([Krauze-Baranowska et al., 2013](#_ENREF_55)) |
|  |  | *S. hulteni* [Flod.](https://en.wikipedia.org/wiki/Bj%C3%B6rn_Floderus) | ([Jeon et al., 2008](#_ENREF_43)) |
|  |  | *S. elbursensis* Boiss. | ([Kompantsev and Shinkarenko, 1975](#_ENREF_54)) |
|  |  | *S. acutifolia* Willd. | ([Zapesochnaya et al., 2002](#_ENREF_116)) |
|  |  | *S. alba* L. | ([Du et al., 2004](#_ENREF_16)) |
|  |  | *S.atrocinerea* Brot.*, S. fragilis* L. and *S.viminalis* L. | ([Ramos et al., 2019](#_ENREF_86)) |
| Naringenin-7-sulfate | (**52**) | *S. integra* [Thunb.](https://en.wikipedia.org/wiki/Carl_Peter_Thunberg) *× S. suchowensis* W.C.Cheng ex G.Zhu | ([Noleto-Dias et al., 2020](#_ENREF_77)) |
| Naringenin-7-*O*-*β*-D-glucoside (prunin) | (**53**) | Willow bark extract (STW 33-I) | ([Freischmidt et al., 2010](#_ENREF_26)) |
|  |  | *S. daphnoides* [Vill.](https://en.wikipedia.org/wiki/Vill.) | ([Krauze-Baranowska et al., 2013](#_ENREF_55)) |
|  |  | *S. acutifolia* Willd. | ([Zapesochnaya et al., 2002](#_ENREF_116)) |
| Naringenin-5-*O*-*β*-D-glucoside (salipurposide) | (**54**) | Willow bark extract (STW 33-I) | ([Freischmidt et al., 2010](#_ENREF_26)) |
|  |  | *S. daphnoides* [Vill.](https://en.wikipedia.org/wiki/Vill.) | ([Krauze-Baranowska et al., 2013](#_ENREF_55)) |
|  |  | *S. elbursensis* Boiss. | ([Kompantsev and Shinkarenko, 1975](#_ENREF_54)) |
|  |  | *S. elbursensis* Boiss. | ([Kompantsev, 1969](#_ENREF_51)) |
|  |  | *S. rubra* Huds. | ([Vinokurov, 1979](#_ENREF_109)) |
|  |  | *S. acutifolia* Willd. | ([Zapesochnaya et al., 2002](#_ENREF_116)) |
| Naringenin-5-*O*-(6ˋˋ-*trans*-p-coumaroyl)-glucoside | (**55**) | Willow bark extract (STW 33-I) | ([Freischmidt et al., 2010](#_ENREF_26)) |
| **D-Dihydroflavonols** | | | |
| Ampelopsin (dihydromyricetin) | (**56**) | willow bark extracts | ([Agnolet et al., 2012](#_ENREF_1)) |
|  |  | *S. hulteni* [Flod.](https://en.wikipedia.org/wiki/Bj%C3%B6rn_Floderus) | ([Jeon et al., 2008](#_ENREF_43)) |
|  |  | *S. caprea* L. | ([Pohjamo et al., 2003](#_ENREF_82)) |
|  |  | *S. pentandra* L. | ([Shao et al., 1989](#_ENREF_95)) |
| Aromadendrin (dihydrokaempferol) | (**57**) | Willow bark extract (STW 33-I) | ([Freischmidt et al., 2010](#_ENREF_26)) |
|  |  | *S. hulteni* [Flod.](https://en.wikipedia.org/wiki/Bj%C3%B6rn_Floderus) | ([Jeon et al., 2008](#_ENREF_43)) |
|  |  | *S. caprea* L. | ([Pohjamo et al., 2003](#_ENREF_82)) |
| Dihydrokaempferol-7-sulfate | (**58**) | *S. integra* [Thunb.](https://en.wikipedia.org/wiki/Carl_Peter_Thunberg) *× S. suchowensis* W.C.Cheng | ([Noleto-Dias et al., 2020](#_ENREF_77)) |
| Taxifolin | (**59**) | Willow bark extract (STW 33-I) | ([Freischmidt et al., 2010](#_ENREF_26)) |
|  |  | *S. caprea* L. | ([Pohjamo et al., 2003](#_ENREF_82)) |
| Taxifolin-7-sulfate | (**60**) | *S. integra* [Thunb.](https://en.wikipedia.org/wiki/Carl_Peter_Thunberg) *× S. suchowensis* W.C.Cheng | ([Noleto-Dias et al., 2020](#_ENREF_77)) |
| 7-*O*-Methyltaxifolin | (**61**) | willow bark extracts | ([Agnolet et al., 2012](#_ENREF_1)) |
| 7-*O*-Methyltaxifolin-  3ˋ-*O*-glucoside | (**62**) | willow bark extracts | ([Agnolet et al., 2012](#_ENREF_1)) |
| **E-Isoflavone** | | | |
| 6,7-Dihydroxy-3ˋ-methoxy-4ˋ,5ˋ-methylenedioxyisoflavone | (**63**) | *S. cheilophila* C.K.Schneid. | ([Shen et al., 2008](#_ENREF_97)) |
| 6,7-Dihydroxy-3ˋ-methoxy-4ˋ,5ˋ-methylenedioxyisoflavone-6-*O*-*β*-D-glucopyranoside | (**64**) |  |  |
| **F- Chalcones** | | | |
| Isosalipurposide | (**65**) | Willow bark extract (STW 33-I) | ([Freischmidt et al., 2010](#_ENREF_26)) |
|  |  | *S. daphnoides* [Vill.](https://en.wikipedia.org/wiki/Vill.) | ([Krauze-Baranowska et al., 2013](#_ENREF_55)) |
|  |  | *S. elbursensis* Boiss. | ([Kompantsev, 1969](#_ENREF_51); [Kompantsev and Shinkarenko, 1975](#_ENREF_54)) |
|  |  | *S. acutifolia* Willd. | ([Zapesochnaya et al., 2002](#_ENREF_116)) |
|  |  | *S. rubra* Huds. | ([Vinokurov, 1979](#_ENREF_109)) |
| 6ˋˋ- *O*-*p*- Coumaroyl isosalipurposide | (**66**) | Willow bark extract (STW 33-I) | ([Freischmidt et al., 2010](#_ENREF_26)) |
|  |  | *S. daphnoides* [Vill.](https://en.wikipedia.org/wiki/Vill.) | ([Krauze-Baranowska et al., 2013](#_ENREF_55)) |
|  |  | *S. acutifolia* Willd. | ([Zapesochnaya et al., 2002](#_ENREF_116)) |
|  |  | *S. rubra* Huds. | ([Vinokurov, 1979](#_ENREF_109)) |
| Naringenin chalcone | (**67**) | *S. daphnoides* [Vill.](https://en.wikipedia.org/wiki/Vill.) | ([Krauze-Baranowska et al., 2013](#_ENREF_55)) |
|  |  | *S. acutifolia* Willd. | ([Zapesochnaya et al., 2002](#_ENREF_116)) |
| **G- Dihydrochalcones** | | | |
| Phlorizin(phloretin-2ˋ-*β*-D-glucopyranoside) | (**68**) | *S. tetrasperma* Roxb. | ([Mostafa et al., 2020](#_ENREF_71)) |
| **H- Flavan-3-ols (catechins and procyanidins)** | | | |
| Catechin | (**69**) | *S. acutifolia* Willd. | ([Wu et al., 2016](#_ENREF_113)) |
|  |  | *S. denticulata* Andersson | ([Semwal et al., 2011](#_ENREF_93)) |
|  |  | *S. hulteni* [Flod.](https://en.wikipedia.org/wiki/Bj%C3%B6rn_Floderus) | ([Jeon et al., 2008](#_ENREF_43)) |
|  |  | *S. acutifolia* Willd. | ([Zapesochnaya et al., 2002](#_ENREF_116)) |
|  |  | *S. purpurea* L. | ([Jürgenliemk et al., 2007](#_ENREF_44)) |
|  |  | *S. alba* L. *x babylonica* L. | ([Kompantsev et al., 1974](#_ENREF_52)) |
|  |  | *S. subserrata* Willd. | ([Hussain et al., 2011](#_ENREF_40); [Tawfeek et al., 2019](#_ENREF_104)) |
|  |  | *S. sieboldiana* [Blume](https://en.wikipedia.org/wiki/Carl_Ludwig_Blume) | ([Hsu et al., 1985](#_ENREF_39)) |
|  |  | *S.atrocinerea* Brot.*, S. fragilis* L. and *S. viminalis* L. | ([Ramos et al., 2019](#_ENREF_86)) |
|  |  | *S. tetrasperma* Roxb. | ([Mostafa et al., 2020](#_ENREF_71)) |
| 3-*O*-(l-hydroxy-6-oxo-2-cyclohexene-l-carboxylic acid ester) of (+)-catechin | (**70**) | *S. purpurea* L. | ([Jürgenliemk et al., 2007](#_ENREF_44)) |
|  |  | *S. sieboldiana* [Blume](https://en.wikipedia.org/wiki/Carl_Ludwig_Blume) | ([Hsu et al., 1985](#_ENREF_39)) |
| 3-*O*-(1,6-dihydroxy-2-cyclohexenc-I-carboxylic acid ester) of (+)-catechin | (**71**) | *S. sieboldiana* [Blume](https://en.wikipedia.org/wiki/Carl_Ludwig_Blume) | ([Hsu et al., 1985](#_ENREF_39)) |
| (epi)Catechin | (**72**) | *S. purpurea* L. | ([Jürgenliemk et al., 2007](#_ENREF_44)) |
|  |  | *S. sieboldiana* [Blume](https://en.wikipedia.org/wiki/Carl_Ludwig_Blume) | ([Hsu et al., 1985](#_ENREF_39)) |
|  |  | *S. tetrasperma* Roxb. | ([Mostafa et al., 2020](#_ENREF_71)) |
| Gallocatechin | (**73**) | *S. purpurea* L. | ([Jürgenliemk et al., 2007](#_ENREF_44)) |
|  |  | *S.alba* L.*x babylonica* L. | ([Kompantsev et al., 1974](#_ENREF_52)) |
|  |  | *S. subserrata* Willd. | ([Tawfeek et al., 2019](#_ENREF_104)) |
|  |  | *S. tetrasperma* Roxb. | ([Mostafa et al., 2020](#_ENREF_71)) |
| (epi)Catechin gallate | (**74**) | *S.alba* L.*x babylonica* L. | ([Kompantsev et al., 1974](#_ENREF_52)) |
| (epi)Gallocatechin gallate | (**75**) | *S.alba* L.*x babylonica* L. | ([Kompantsev et al., 1974](#_ENREF_52)) |
| (epi)Gallocatechindigallate | (**76**) | *S. tetrasperma* Roxb. | ([Mostafa et al., 2020](#_ENREF_71)) |
| Procyanidin B1[epicatechin-(4*ß*→8)-catechin] | (**77**) | *S. purpurea* L. | ([Jürgenliemk et al., 2007](#_ENREF_44)) |
|  |  | *Salix purpurea* L.*, S. fragilis* L. *and S. alba* L. | ([Pobłocka-Olech and Krauze-Baranowska, 2008](#_ENREF_81)) |
|  |  | *S. sieboldiana* [Blume](https://en.wikipedia.org/wiki/Carl_Ludwig_Blume) | ([Hsu et al., 1985](#_ENREF_39)) |
|  |  | *S. daphnoides* [Vill.](https://en.wikipedia.org/wiki/Vill.) | ([Wiesneth, 2019](#_ENREF_112)) |
|  |  | *S.atrocinerea* Brot.*, S. fragilis* L. and *S.*  *viminalis* L. | ([Ramos et al., 2019](#_ENREF_86)) |
|  |  | *S. tetrasperma* Roxb. | ([Mostafa et al., 2020](#_ENREF_71)) |
| 3ˋ-*O*-(l-Hydroxy-6-oxo-2-cyclohexene-l-carboxylic acid ester) of procyanidin B_1_ | (**78**) | *S. sieboldiana* [Blume](https://en.wikipedia.org/wiki/Carl_Ludwig_Blume) | ([Hsu et al., 1985](#_ENREF_39)) |
| Procyanidin B2[epicatechin-(4*ß*→8)- epicatechin] | (**79**) | *Salix purpurea* L.*, S. fragilis* L. *and S. alba* L. | ([Pobłocka-Olech and Krauze-Baranowska, 2008](#_ENREF_81)) |
|  |  | *S. daphnoides* [Vill.](https://en.wikipedia.org/wiki/Vill.) | ([Wiesneth, 2019](#_ENREF_112)) |
|  |  | *S.atrocinerea* Brot.*, S. fragilis* L. and *S. viminalis* L. | ([Ramos et al., 2019](#_ENREF_86)) |
| Procyanidin B_3_[catechin-(4*α*→8)-catechin] | (**80**) | *S. purpurea* L. | ([Jürgenliemk et al., 2007](#_ENREF_44)) |
|  |  | *S. sieboldiana* [Blume](https://en.wikipedia.org/wiki/Carl_Ludwig_Blume) | ([Hsu et al., 1985](#_ENREF_39)) |
|  |  | *S. daphnoides* [Vill.](https://en.wikipedia.org/wiki/Vill.) | ([Wiesneth, 2019](#_ENREF_112)) |
|  |  | *S.atrocinerea* Brot.*, S. fragilis* L. and *S. viminalis* L. | ([Ramos et al., 2019](#_ENREF_86)) |
| 3ˋ-*O*-(l-hydroxy-6-oxo-2-cyclohexene-l-carboxylic acid ester) of Procyanidin B3 | (**81**) | *S. sieboldiana* [Blume](https://en.wikipedia.org/wiki/Carl_Ludwig_Blume) | ([Hsu et al., 1985](#_ENREF_39)) |
| Procyanidin B4 [catechin-(4α→8)-epicatechin] | (**82**) | *S. daphnoides* [Vill.](https://en.wikipedia.org/wiki/Vill.) | ([Wiesneth, 2019](#_ENREF_112)) |
| Procyanidin B5 | (**83**) |  |  |
| Procyanidins B6 | (**84**) | *S. sieboldiana* [Blume](https://en.wikipedia.org/wiki/Carl_Ludwig_Blume) | ([Hsu et al., 1985](#_ENREF_39)) |
| Procyanidins B7 | (**85**) | *S. sieboldiana* [Blume](https://en.wikipedia.org/wiki/Carl_Ludwig_Blume) | ([Hsu et al., 1985](#_ENREF_39)) |
|  |  | *S. daphnoides* [Vill.](https://en.wikipedia.org/wiki/Vill.) | ([Wiesneth, 2019](#_ENREF_112)) |
| Procyandin C1 [epicatechin-(4β→8)-epicatechin-(4β→8)-epicatechin] | (**86**) | *S. daphnoides* [Vill.](https://en.wikipedia.org/wiki/Vill.) | ([Wiesneth, 2019](#_ENREF_112)) |
| Trimeric procyanidin epicatechin-(4*ß*→8)-catechin-(4*α*→8)-catechin | (**87**) | *S. purpurea* L. | ([Jürgenliemk et al., 2007](#_ENREF_44)) |
|  |  | *S. sieboldiana* [Blume](https://en.wikipedia.org/wiki/Carl_Ludwig_Blume) | ([Hsu et al., 1985](#_ENREF_39)) |
| 3"-*O*-(l-Hydroxy-6-oxo-2-cyclohexene-l-carboxylic acid ester) of procyanidin trimer | (**88**) | *S. sieboldiana* [Blume](https://en.wikipedia.org/wiki/Carl_Ludwig_Blume) | ([Hsu et al., 1985](#_ENREF_39)) |
| Trimeric procyanidin epicatechin-(4ß→8)-epicatechin-(4ß→8)-catechin | (**89**) | *S. purpurea* L. | ([Jürgenliemk et al., 2007](#_ENREF_44)) |
|  |  | *S. sieboldiana* [Blume](https://en.wikipedia.org/wiki/Carl_Ludwig_Blume) | ([Hsu et al., 1985](#_ENREF_39)) |
|  |  | *S. daphnoides* [Vill.](https://en.wikipedia.org/wiki/Vill.) | ([Wiesneth, 2019](#_ENREF_112)) |
| Catechin-(4α→8)-epicatechin-(4β→8)-catechin | (**90**) | *S. daphnoides* [Vill.](https://en.wikipedia.org/wiki/Vill.) | ([Wiesneth, 2019](#_ENREF_112)) |
| (epi)Gallocatechin-(epi)catechin-(epi)catechin | (**91**) | *S. tetrasperma* Roxb. | ([Mostafa et al., 2020](#_ENREF_71)) |
| Epicatechin-(4β→8)-epicatechin-(4β→8)-epicatechin-(4β→8)-catechin | (**92**) | *S. daphnoides* [Vill.](https://en.wikipedia.org/wiki/Vill.) | ([Wiesneth, 2019](#_ENREF_112)) |
| **I-Anthocyanins** | | | |
| Cyanidin-3-glucoside | (**93**) | *S. purpurea* L. | ([Bridle et al., 1973](#_ENREF_11)) |
|  |  | *S. daphnoides* [Vill.](https://en.wikipedia.org/wiki/Vill.)*, S. alba* L.*, S. phylicifolia* [L.](https://en.wikipedia.org/wiki/Carl_Linnaeus)*, S. nigricans* Sm.*, S. calodendron* Wimm., *S. viminalis* L.*, S. triandra* L. *and S. amygdalina* L. | ([Bridle et al., 1970](#_ENREF_10)) |
| Delphinidin-3-glucoside | (**94**) | *S. purpurea* L. | ([Bridle et al., 1973](#_ENREF_11)) |
|  |  | *S. phylicifolia* [L.](https://en.wikipedia.org/wiki/Carl_Linnaeus)*, S. nigricans* Sm.*, S. calodendron* Wimm.*, S. viminalis* L.*, S. triandra* L. *and S. amygdalina* L. | ([Bridle et al., 1970](#_ENREF_10)) |
| Petunidin-3-glucoside | (**95**) | S. *purpurea* L. | ([Bridle et al., 1973](#_ENREF_11)) |
|  |  | *S. triandra* L. and *S. amygdalina* L. | ([Bridle et al., 1970](#_ENREF_10)) |

| **Supplementary TABLE S2.** Reported phenolic glycosides from genus *Salix*. | | | |
| --- | --- | --- | --- |
| **Compound** | | **Species** | **References** |
| **Name** | **No.** |  |  |
| Acmophyllin A | (**96**) | *S. acmophylla* Boiss. | ([Shah et al., 2016](#_ENREF_94)) |
| Acmophyllin B | (**97**) |  |  |
| Acutifoliside | (**98**) | *S. acutifolia* Willd. | ([Wu et al., 2016](#_ENREF_113)) |
| Acutifoliside glucoside linoleic acid | (**99**) | *S. tetrasperma* Roxb. | ([Sobeh et al., 2019](#_ENREF_99)) |
| 2-[(benzoyloxy)methyl]-4-hydroxyphenyl *β*-D-glucopyranoside 3-benzoate | (**100**) | *S. babylonica* L. | ([Singh et al., 2017](#_ENREF_98)) |
| Chaenomeloidin | (**101**) | *S. chaenomeloides*  Kimura | ([Mizuno et al., 1991](#_ENREF_70)) |
| Chavicol-*α*-L-arabinofuranosyl-(1→6)-*β*-D- glucopyranoside | (**102**) | *S. triandra* L.x*dasyclados* Wimmer | ([Noleto-Dias et al., 2019](#_ENREF_79)) |
| Chavicol-*α*-L-arabinopyranosyl -(1→6)-β-D- glucopyranoside | (**103**) |  |  |
| Chavicol-*β*-D-apiofuranosyl -(1→6)-*β*-D- glucopyranoside | (**104**) |  |  |
| Chavicol-glucoside | (**105**) |  |  |
| Chavicol-rutinoside | (**106**) |  |  |
| Cochinchiside A | (**107**) | *S. glandulosa* Seemen. | ([Kim et al., 2015](#_ENREF_49)) |
| Coumaroylgalloyl glucose | (**108**) | *S. tetrasperma* Roxb. | ([Sobeh et al., 2019](#_ENREF_99)) |
| Coumaric acid galloyl pentoside | (**109**) | *S. tetrasperma* Roxb. | ([Mostafa et al., 2020](#_ENREF_71)) |
| *p*-Coumaroyl-β-D-glucoside | (**110**) | *S. hulteni* [Flod.](https://en.wikipedia.org/wiki/Bj%C3%B6rn_Floderus) | ([Jeon et al., 2008](#_ENREF_43)) |
|  |  | *S. triandra* L. *x dasyclados* Wimmer | ([Noleto-Dias et al., 2019](#_ENREF_79)) |
| Eugenol-1-*O*-*β*-D-glucopyranoside | (**111**) | *S. denticulata* Andersson | ([Rawat et al., 2009](#_ENREF_87)) |
| Feruloyl glucose | (**112**) | *S. cheilophila* C.K.Schneid. | ([Shen et al., 2008](#_ENREF_97)) |
| Fragilin (6`-*O*-acetylsalicin) | (**113**) | *S. pentandroides* A.K.Skvortsov | ([Kompantsev and Shinkarenko, 1973](#_ENREF_53)) |
|  |  | *S. glandulosa* Seemen. | ([Kim et al., 2015](#_ENREF_49)) |
| Gallic acid glucoside | (**114**) | *S. tetrasperma* Roxb. | ([Mostafa et al., 2020](#_ENREF_71)) |
| Gallic acid glucuronide-glucoside | (**115**) |  |  |
| *trans*-Glanduloidin A | (**116**) | *S. glandulosa* Seemen. | ([Kim et al., 2014](#_ENREF_48)) |
| *cis*-Glanduloidin A | (**117**) |  |  |
| *trans*-Glanduloidin B | (**118**) |  |  |
| *cis*-Glanduloidin B | (**119**) |  |  |
| Glanduloidin C | (**120**) |  |  |
| *trans*-Glanduloidin D | (**121**) |  |  |
| *cis*-Glanduloidin D | (**122**) |  |  |
| *cis*-Grandidentatin | (**123**) |  |  |
| Grandidentatin | (**124**) |  |  |
| *trans*-Glanduloidin E | (**125**) |  |  |
| *cis*-Glanduloidin E | (**126**) |  |  |
| *trans*-Glanduloidin F | (**127**) |  |  |
| *cis*-Glanduloidin F | (**128**) |  |  |
| *p*-Hydroxy benzoyl galloyl glucose | (**129**) | *S. tetrasperma* Roxb. | ([Mostafa et al., 2020](#_ENREF_71)) |
| *p* -Hydroxy benzoyl protocatechuic acid glucose | (**130**) | *S. tetrasperma* Roxb. | ([Mostafa et al., 2020](#_ENREF_71)) |
| *p* -Hydroxybenzylacetone-β-D- glucoside | (**131**) | *S.triandra* L. *x. dasyclados* Wimmer | ([Noleto-Dias et al., 2019](#_ENREF_79)) |
| 4-(Hydroxymethyl) phenyl *β*-D-glucopyranoside | (**132**) | *S. babylonica* L. | ([Singh et al., 2017](#_ENREF_98)) |
| Lasiandrin | (**133**) | *S. lasiandra* | ([Reichardt et al., 1992](#_ENREF_88)) |
| Leonuriside A | (**134**) | *S. matsudana* Koidz. | ([Li et al., 2008](#_ENREF_61)) |
| Piceoside (picein) | (**135**) | *S. matsudana* Koidz. | ([Li et al., 2008](#_ENREF_61)) |
|  |  | *S. triandra* L. *x dasyclados* Wimmer | ([Noleto-Dias et al., 2019](#_ENREF_79)) |
|  |  | *S. hulteni* [Flod.](https://en.wikipedia.org/wiki/Bj%C3%B6rn_Floderus) | ([Jeon et al., 2008](#_ENREF_43)) |
|  |  | *S.atrocinerea* Brot.*, S. fragilis* L. and *S.viminalis* L. | ([Ramos et al., 2019](#_ENREF_86)) |
| Populin | (**136**) | *S. fragilis* L. | ([Binns et al., 1968](#_ENREF_9)) |
| Populoside B | (**137**) | Willow bark extract (STW 33-I) | ([Freischmidt et al., 2010](#_ENREF_26)) |
| Protocatechuic acid 3-*O*-hexoside | (**138**) | *S. tetrasperma* Roxb. | ([Sobeh et al., 2019](#_ENREF_99)) |
| Sachaliside 1 | (**139**) | *S. hulteni* [Flod.](https://en.wikipedia.org/wiki/Bj%C3%B6rn_Floderus) | ([Jeon et al., 2008](#_ENREF_43)) |
|  |  | *S. sachalinensis* F.Schmidt | ([Mizuno et al., 1990](#_ENREF_68)) |
| Sachaliside 2 | (**140**) | *S. sachalinensis* F.Schmidt | ([Mizuno et al., 1990](#_ENREF_68)) |
| Salicin | (**141**) | *S. acutifolia* Willd. | ([Wu et al., 2016](#_ENREF_113)) |
|  |  | *S. martiana* Leyb. | ([Fernandes et al., 2009](#_ENREF_25)) |
|  |  | *S. chaenomeloides*  Kimura | ([Mizuno et al., 1991](#_ENREF_70)) |
|  |  | *S. pentandroides* A.K.Skvortsov | ([Kompantsev and Shinkarenko, 1973](#_ENREF_53)) |
|  |  | *S. glandulosa* Seemen. | ([Kim et al., 2015](#_ENREF_49)) |
|  |  | *S. acmophylla* Boiss. | ([Shah et al., 2016](#_ENREF_94)) |
|  |  | *S. babylonica* L. | ([Khatoon et al., 1988](#_ENREF_47)) |
|  |  | *S.tetrasperma* Roxb. | ([El-Shazly et al., 2012](#_ENREF_19); [Sobeh et al., 2019](#_ENREF_99)) |
|  |  | *S. subserrata* Willd. | ([Tawfeek et al., 2019](#_ENREF_104)) |
| 2`-*O*-Acetylsalicin | (**142**) | *S. glandulosa* Seemen. | ([Kim et al., 2015](#_ENREF_49)) |
|  |  | *S. lasiandra* | ([Reichardt et al., 1992](#_ENREF_88)) |
|  |  | *S. pentandra* L. | ([Shao et al., 1989](#_ENREF_95)) |
| 3`-*O*-Acetylsalicin | (**143**) | *S. glandulosa* Seemen. | ([Kim et al., 2015](#_ENREF_49)) |
| 2`-*O*-(*E*)-*p*-Coumaroylsalicin | (**144**) | *S. glandulosa* Seemen. | ([Kim et al., 2015](#_ENREF_49)) |
|  |  | *S. acmophylla* Boiss. | ([Shah et al., 2016](#_ENREF_94)) |
|  |  | *S. tetrasperma* Roxb. | ([El-Shazly et al., 2012](#_ENREF_19)) |
| 2`-*O*-(*Z*)-*p*-Coumaroylsalicin | (**145**) | *S. glandulosa* Seemen. | ([Kim et al., 2015](#_ENREF_49)) |
| 6`-*O*-(*E*)-*p*-Coumaroylsalicin | (**146**) |  |  |
| 6`-*O*-(*Z*)-*p*-Coumaroylsalicin | (**147**) |  |  |
| Coumaroyl dihydrobenzoylsalicin | (**148**) | *S. tetrasperma* Roxb. | ([Sobeh et al., 2019](#_ENREF_99); [Mostafa et al., 2020](#_ENREF_71)) |
| Cinnamoyl salicin | (**149**) | *S. tetrasperma* Roxb. | ([Sobeh et al., 2019](#_ENREF_99)) |
| Dihydrocinnamoylsalicin | (**150**) |  |  |
| Salicin malate | (**151**) |  |  |
| Salicin-7-sulfate | (**152**) | *S. koriyanagi* Kimura. | ([Noleto-Dias et al., 2018](#_ENREF_78)) |
| Salicyloylsalicin | (**153**) | *S. acmophylla* Boiss. | ([Shah et al., 2016](#_ENREF_94)) |
| Salicortin | (**154**) | *S. acutifolia* Willd. | ([Zapesochnaya et al., 2002](#_ENREF_116); [Wu et al., 2016](#_ENREF_113)) |
|  |  | *S. glandulosa* Seemen. | ([Kim et al., 2015](#_ENREF_49)) |
|  |  | *S. pseudo-lasiogyne* H.Lév. | ([Lee et al., 2013](#_ENREF_58)) |
|  |  | *S. lasiandra* | ([Reichardt et al., 1992](#_ENREF_88)) |
|  |  | *S. tetrasperma* Roxb. | ([Mostafa et al., 2020](#_ENREF_71)) |
| 2`-*O*-Acetylsalicortin | (**155**) | *S. glandulosa* Seemen. | ([Kim et al., 2015](#_ENREF_49)) |
|  |  | *S. pseudo-lasiogyne* H.Lév. | ([Lee et al., 2013](#_ENREF_58)) |
|  |  | *S. lasiandra* | ([Reichardt et al., 1992](#_ENREF_88)) |
| 3`-*O*-Acetylsalicortin | (**156**) | *S. glandulosa* Seemen. | ([Kim et al., 2015](#_ENREF_49)) |
|  |  | *S. pseudo-lasiogyne* H.Lév. | ([Lee et al., 2013](#_ENREF_58)) |
| 6`-*O*-Acetylsalicortin | (**157**) | *S. glandulosa* Seemen. | ([Kim et al., 2015](#_ENREF_49)) |
|  |  | *S. pseudo-lasiogyne* H.Lév. | ([Lee et al., 2013](#_ENREF_58)) |
| 2`,6`-*O*-Acetylsalicortin | (**158**) | *S. pseudo-lasiogyne* H.Lév. | ([Lee et al., 2013](#_ENREF_58)) |
| 2ˋCinnamoylsalicortin | (**159**) | *S. sericea* Marsh. | ([Nichols-Orians et al., 1992](#_ENREF_76)) |
| Salidroside | (**160**) | *S. babylonica* L. | ([Binns et al., 1968](#_ENREF_9)) |
|  |  | *S. triandra* L. *x dasyclados* Wimmer | ([Noleto-Dias et al., 2019](#_ENREF_79)) |
| Saliglandin | (**161**) | *S. glandulosa* Seemen. | ([Kim et al., 2015](#_ENREF_49)) |
| Sinapic acid 3-*O*-glucoside | (**162**) | *S. tetrasperma* Roxb. | ([Sobeh et al., 2019](#_ENREF_99)) |
| Syringin | (**163**) | *S. acutifolia* Willd. | ([Zapesochnaya et al., 2002](#_ENREF_116)) |
| Tremuloidin | (**164**) | *S. acutifolia* Willd. | ([Wu et al., 2016](#_ENREF_113)) |
|  |  | *S. chaenomeloides*  Kimura | ([Mizuno et al., 1991](#_ENREF_70)) |
|  |  | *S. glandulosa* Seemen. | ([Kim et al., 2015](#_ENREF_49)) |
|  |  | *S. acmophylla* Boiss. | ([Shah et al., 2016](#_ENREF_94)) |
|  |  | *S. babylonica* L. | ([Zeid, 2006](#_ENREF_118)) |
|  |  | *S. tetrasperma* Roxb. | ([El-Shazly et al., 2012](#_ENREF_19)) |
| Salicyloyltremuloidin | (**165**) | *S. chaenomeloides*  Kimura | ([Mizuno et al., 1991](#_ENREF_70)) |
| Tremulacin | (**166**) | *S. chaenomeloides*  Kimura | ([Mizuno et al., 1991](#_ENREF_70)) |
|  |  | *S. acutifolia* Willd. | ([Zapesochnaya et al., 2002](#_ENREF_116)) |
|  |  | *S. glandulosa* Seemen. | ([Kim et al., 2015](#_ENREF_49)) |
|  |  | *S.tetrasperma* Roxb. | ([El-Shazly et al., 2012](#_ENREF_19); [Sobeh et al., 2019](#_ENREF_99); [Mostafa et al., 2020](#_ENREF_71)) |
| Trichocarposide | (**167**) | *S. martiana* Leyb. | ([Fernandes et al., 2009](#_ENREF_25)) |
|  |  | *S. tetrasperma* Roxb. | ([Mostafa et al., 2020](#_ENREF_71)) |
| Triandrin | (**168**) | *S. acutifolia* Willd. | ([Zapesochnaya et al., 2002](#_ENREF_116)) |
|  |  | *S. alba* L. *x S. babylonica* L. | ([Kompantsev et al., 1974](#_ENREF_52)) |
|  |  | *S. triandra* L. *x dasyclados* Wimmer | ([Noleto-Dias et al., 2019](#_ENREF_79)) |
|  |  | *S. subserrata* Willd. | ([Tawfeek et al., 2019](#_ENREF_104)) |
| Trichocarpin | (**169**) | *S. babylonica* L. | ([Khatoon et al., 1988](#_ENREF_47); [Zeid, 2006](#_ENREF_118)) |
| 2ˋ-*O*-Acetyltrichocarpin | (**170**) | *S. babylonica* L. | ([Khatoon et al., 1988](#_ENREF_47)) |
| Vimalin | (**171**) | *S. babylonica* L. | ([Binns et al., 1968](#_ENREF_9)) |

| **Supplementary TABLE S3.** Reported non-phenolic glycosides from genus *Salix*. | | | | |
| --- | --- | --- | --- | --- |
| **Compound** | | **Species** | | **References** |
| **Name** | **No.** |  |  |  |
| Benzyl-6-*O-β-D-*glucopranosyl-1,6-dihydroxy-2,5-cyclohexadienyl carboxylate | (**172**) | | *S.arbusculoides* Andersson | ([Evans et al., 1995](#_ENREF_23)) |
| Benzyl *1* -*O*-*β*-*D*-gluropyranosy- 1 -hydroxy-6-*oxo-2-cyclohexenyl* carboxylate | (**173**) | |  |  |
| Benzyl-*β*-D-apiofuranosyl-(1→6)- *β* -D- glucopyranoside | (**174**) | | *S.triandra* L.x*dasyclados* Wimmer | ([Noleto-Dias et al., 2019](#_ENREF_79)) |
| Benzyl- *β* -D-xylopyranosyl-(1→6)- *β* -D- glucopyranoside | (**175**) | |  |  |
| Cinnamrutinose A | (**176**) | |  |  |
| Dihydrorosarin | (**177**) | |  |  |
| Dihydrorosavin | (**178**) | |  |  |
| (1R,2R)-*trans*-1,2-Cyclohexanediol-1-*O*-*β*-D-glucopyranoside | (**179**) | | *S. glandulosa* Seemen. | ([Kim et al., 2014](#_ENREF_48)) |
| (1S,2S)-*trans*-1,2-Cyclohexanediol-1-*O*-*β*-D-glucopyranoside | (**180**) | |  |  |
| Grandidentin (1,2- cyclohexanediol-*β*-D-glucopyranoside) | (**181**) | | *S. purpurea* L. | ([Pearl and Darling, 1970](#_ENREF_80)) |
| 2-Phenylethyl-*α*-L-arabinofuranosyl -(1→6)-*β*-D- glucopyranoside | (**182**) | | *S.triandra* L.*x dasyclados* Wimmer | ([Noleto-Dias et al., 2019](#_ENREF_79)) |
| 2-Phenylethyl-*α*-L-arabinopyranosyl -(1→6)-*β*-D- glucopyranoside | (**183**) | |  |  |
| 2-Phenylethyl-*β*-D-apiofuranosyl -(1→6)-*β*-D- glucopyranoside | (**184**) | |  |  |
| Phenylpropanol-*β*-D- apiofuranosyl -(1→6)-*β*-D- glucopyranoside | (**185**) | |  |  |
| Rosarin | (**186**) | |  |  |
| Rosin | (**187**) | |  |  |
| Rosavin | (**188**) | |  |  |

| **Supplementary TABLE S4.** Reported organic acids, especially phenolic acids from the genus *Salix*. | | | |
| --- | --- | --- | --- |
| **Compound** | | **Species** | **References** |
| **Name** | **No.** |  |  |
| (±)-(2*E*,4*E*)-Abscisic acid | (**189**) | *S. alba* L.*, S. caprea* L. | ([Tuberoso et al., 2011](#_ENREF_106)) |
| (±)-(2*Z*,4*E*)-Abscisic acid | (**190**) |  |  |
| 1,2-Benzenedicarboxylic acid, bis (2-ethylhexyl) ester | (**191**) | *S. subserrata* Willd. | ([Hussain et al., 2011](#_ENREF_40)) |
| Benzoic acid | (**192**) | *S. purpurea* L. *and S. alba* L. | ([Agnolet et al., 2012](#_ENREF_1)) |
| *p*-Hydroxybenzoic acid | (**193**) | *S. purpurea* L. *and S. alba* L. | ([Agnolet et al., 2012](#_ENREF_1)) |
| *p*-Methoxybenzoic acid | (**194**) | *S. purpurea* L. *and S. alba* L. | ([Agnolet et al., 2012](#_ENREF_1)) |
| Vanillic acid | (**195**) | *S. caprea* L. | ([Pohjamo et al., 2003](#_ENREF_82)) |
| Caffeic acid | (**196**) | *S. cheilophila* C.K.Schneid. | ([Shen et al., 2008](#_ENREF_97)) |
| Caffeoylmalic acid | (**197**) | *S. tetrasperma* Roxb. | ([Sobeh et al., 2019](#_ENREF_99)) |
| Cinnamic acid | (**198**) | *S. purpurea* L.*and S. alba* L. | ([Agnolet et al., 2012](#_ENREF_1)) |
| *trans*-*p-*Methoxycinnamic acid | (**199**) | *S. purpurea* L. *and S. alba* L. | ([Agnolet et al., 2012](#_ENREF_1)) |
| *cis*-*p-*Methoxycinnamic acid | (**200**) | *S. purpurea* L. *and S. alba* L. | ([Agnolet et al., 2012](#_ENREF_1)) |
| *p*-Coumaric acid | (**201**) | *S. rubra* Huds. | ([Vinokurov, 1979](#_ENREF_109)) |
| Coumaroylquinic acid | (**202**) | *S. tetrasperma* Roxb. | ([Sobeh et al., 2019](#_ENREF_99)) |
| Coumaroyl malic acid | (**203**) |  |  |
| Eicosanoic acid | (**204**) | *S. tetrasperma* Roxb. | ([Mostafa et al., 2020](#_ENREF_71)) |
| Hydroxy-octadecadienoic acid | (**205**) | *S. tetrasperma* Roxb. | ([Mostafa et al., 2020](#_ENREF_71)) |
| Hydroxy-octadecatrienoic acid | (**206**) |  |  |
| Kynurenic acid | (**207**) | *S. alba* L. *& S. caprea* L. | ([Tuberoso et al., 2011](#_ENREF_106)) |
| Malic acid | (**208**) | *S. tetrasperma* Roxb. | ([Mostafa et al., 2020](#_ENREF_71)) |
| Phloretic acid | (**209**) | *S. tetrasperma* Roxb. | ([Sobeh et al., 2019](#_ENREF_99)) |
| Pipecolic acid | (**210**) | *S. calodendron* Wimm.*, S. cinerea* L.*, S. decipiens* Hoffm.  *, S. caprea* L*., S. nigricans* Sm.*, S triandra* L. *and S.viminalis* L. | ([Binns et al., 1968](#_ENREF_9)) |
| 5-Hydroxy-Pipecolic acid | (**211**) | *S. alba* L.*&S.fragilis* L. | ([Binns et al., 1968](#_ENREF_9)) |
| Propyl acetate | (**212**) | *S. subserrata* Willd. | ([Hussain et al., 2011](#_ENREF_40)) |
| Methyl 1-hydroxy-6-oxocyclohex-2-enecarboxylate | (**213**) | *S. subserrata* Willd. | ([Hussain et al., 2011](#_ENREF_40)) |
| Ethyl -1-hydroxy-6-oxocyclohex-2-enecarboxylate | (**214**) | *S. purpurea* L. *and S. alba* L. | ([Agnolet et al., 2012](#_ENREF_1)) |
| Quinic acid | (**215**) | *S. tetrasperma* Roxb. | ([Mostafa et al., 2020](#_ENREF_71)) |
| Salicylic acid | (**216**) | *S.atrocinerea* Brot.*, S. fragilis* L. and *S. viminalis* L. | ([Ramos et al., 2019](#_ENREF_86)) |

| **Supplementary TABLE S5.** Reported simple phenolics from genus *Salix*. | | | |
| --- | --- | --- | --- |
| **Compound** | | **Species** | **References** |
| **Name** | **No.** |  |  |
| Acylsaligenin (salicyl-HCH) | (**217**) | *S. acutifolia* Willd. | ([Zapesochnaya et al., 2002](#_ENREF_116)) |
| Aucuparin | (**218**) | *S. caprea* L. | ([Malterud and Dugstad, 1985](#_ENREF_66)) |
| Methoxyaucuparin | (**219**) |  |  |
| Catechol | (**220**) | Willow bark extract (STW 33-I) | ([Freischmidt et al., 2010](#_ENREF_26)) |
|  |  | *S. capensis* | ([Masika et al., 2005](#_ENREF_67)) |
|  |  | *S. rubra* Huds. | ([Vinokurov, 1979](#_ENREF_109)) |
|  |  | *S. tetrasperma* Roxb. | ([El-Shazly et al., 2012](#_ENREF_19)) |
|  |  | *S. subserrata* Willd. | ([Hussain et al., 2011](#_ENREF_40)) |
| Coniferyl alcohol | (**221**) | *S. caprea* L. | ([Pohjamo et al., 2003](#_ENREF_82)) |
| *p*-Coumaryl alcohol | (**222**) | *S. caprea* L. | ([Pohjamo et al., 2003](#_ENREF_82)) |
| 4,2'-Dihydroxy-3,5dimethoxybiphenyl | (**223**) | *Salix caprea* L. | ([Malterud and Dugstad, 1985](#_ENREF_66)) |
| 2,6-Dihydroxy-4-methoxy acetophenone | (**224**) | *S. denticulata* Andersson | ([Rawat et al., 2009](#_ENREF_87)) |
| 5,7-Dihydroxychromen-4-one | (**225**) | *S. alba* L. | ([Du et al., 2004](#_ENREF_16)) |
| 4-Hydroxyacetophenone (piceol) | (**226**) | *S. hulteni* [Flod.](https://en.wikipedia.org/wiki/Bj%C3%B6rn_Floderus) | ([Jeon et al., 2008](#_ENREF_43)) |
|  |  | *S.atrocinerea* Brot.*, S. fragilis* L. and *S.viminalis* L. | ([Ramos et al., 2019](#_ENREF_86)) |
| 5-(Hydroxymethyl) furfural (nonphenolic) | (**227**) | *S. alba* L. *and S. caprea* L. | ([Tuberoso et al., 2011](#_ENREF_106)) |
| Saligenin (salicyl alcohol) | (**228**) | *S. capensis* | ([Masika et al., 2005](#_ENREF_67)) |
|  |  | *S. acutifolia* Willd. | ([Zapesochnaya et al., 2002](#_ENREF_116)) |
|  |  | *S. subserrata* Willd. | ([Hussain et al., 2011](#_ENREF_40)) |
|  |  | *S. caprea* L. | ([Ahmed et al., 2017](#_ENREF_2)) |
| Sinapylaldehyde | (**229**) | *S. caprea* L. | ([Pohjamo et al., 2003](#_ENREF_82)) |

| **Supplementary TABLE S6.** Reported sterols and terpenes from genus *Salix*. | | | |
| --- | --- | --- | --- |
| **Compound** | | **Species** | **References** |
| **Name** | **No.** |  |  |
| **A-Sterols / triterpenes** | | | |
| *ß*-Amyrin | (**230**) | *S. tetrasperma* Roxb. | ([El-Shazly et al., 2012](#_ENREF_19)) |
| Campesterol | (**231**) | *S. subserrata* Willd. | ([Balbaa et al., 1979](#_ENREF_8)) |
| Friedelin | (**232**) | *S. tetrasperma* Roxb. | ([El-Shazly et al., 2012](#_ENREF_19)) |
| 3*β*-Friedelinol | (**233**) | *S. tetrasperma* Roxb. | ([El-Shazly et al., 2012](#_ENREF_19)) |
| Porusterone I | (**234**) | *S. cheilophila* C.K.Schneid. | ([Shen et al., 2008](#_ENREF_97)) |
| *ß* -Sitosterol | (**235**) | *S. cheilophila* C.K.Schneid. | ([Shen et al., 2008](#_ENREF_97)) |
|  |  | *S. denticulata* Andersson | ([Rawat et al., 2009](#_ENREF_87)) |
|  |  | *S. babylonica* L. | ([Singh et al., 2017](#_ENREF_98)) |
|  |  | *S. tetrasperma* Roxb. | ([El-Shazly et al., 2012](#_ENREF_19)) |
|  |  | *S. subserrata* Willd. | ([Hussain et al., 2011](#_ENREF_40)) |
| *β*-Sitosterolacetate | (**236**) | *S. tetrasperma* Roxb. | ([El-Shazly et al., 2012](#_ENREF_19)) |
| *β*-Sitosterol-*O*-glucoside | (**237**) | *S. tetrasperma* Roxb. | ([El-Shazly et al., 2012](#_ENREF_19)) |
|  |  | *S. subserrata* Willd. | ([Hussain et al., 2011](#_ENREF_40)) |
| Sitosterol glucoside linoleic acid | (**238**) | *S. tetrasperma* Roxb. | ([Sobeh et al., 2019](#_ENREF_99)) |
| Stigmast-4-en-3*ß* -ol-6-one | (**239**) | *S. cheilophila* C.K.Schneid. | ([Shen et al., 2008](#_ENREF_97)) |
| Stigmast-5-en-3 *ß*-ol-7-one | (**240**) | *S. cheilophila* C.K.Schneid. | ([Shen et al., 2008](#_ENREF_97)) |
| Stigmasterol | (**241**) | *S. subserrata* Willd. | ([Balbaa et al., 1979](#_ENREF_8)) |
| **B- Diterpenes** | | | |
| Shaliuin I (phytane diterpene) | (**242**) | *S. cheilophila* C.K.Schneid. | ([Shen et al., 2008](#_ENREF_97)) |
| Shaliuin II (phytane diterpene) | (**243**) |  |  |
| Shaliuin III (phytane diterpene) | (**244**) |  |  |
| Hanliuin IV (phytane diterpene) | (**245**) |  |  |
| 8*ß* ,19-Dihydroxy-3-oxopimar-15-ene (pimarane diterpene) | (**246**) |  |  |

| **Supplementary TABLE S7.** Reported fatty acids and volatiles from the genus *Salix* | | | |
| --- | --- | --- | --- |
| **Compound** | | **Species** | **References** |
| **Name** | **No.** |  |  |
| 4-Acetoxy-3-methoxycinnamic acid | (**254**) | *S. alba* L. | ([Zarger et al., 2014](#_ENREF_117)) |
| Acetyl monoglyceride | (**255**) |  |  |
| 3-Allyl-2-methoxyphenol | (**256**) |  |  |
| 1-Amino-4-methylpiperazine | (**257**) |  |  |
| Benzyl isopentyl ether | (**258**) |  |  |
| 1,2,3-Benzenetriol, triacetate | (**259**) |  |  |
| Bis (2-ethylhexyl) adipate | (**260**) | *S. babylonica* L. | ([González-Alamilla et al., 2019](#_ENREF_30)) |
| 2-Butyl-octanol | (**261**) | *S. caprea* L. | ([Ahmed et al., 2017](#_ENREF_2)) |
| 2-Butynyl *p*-toluenesulfonate | (**262**) | *S. alba* L. | ([Zarger et al., 2014](#_ENREF_117)) |
| Carvone | (**263**) | *S. aegyptiaca* L. | ([Karimi et al., 2011](#_ENREF_46)) |
| 3*α*-Cholesterol methyl ether | (**264**) | *S. alba* L. | ([Zarger et al., 2014](#_ENREF_117)) |
| Citronellol | (**265**) | *S. aegyptiaca* L. | ([Karimi et al., 2011](#_ENREF_46)) |
| 1,2-Cyclohexanediol | (**266**) | *S. alba* L. | ([Zarger et al., 2014](#_ENREF_117)) |
|  |  | *S. babylonica* L. | ([González-Alamilla et al., 2019](#_ENREF_30)) |
| *β*-Damascenone | (**267**) | *S. caprea* L. | ([Ahmed et al., 2017](#_ENREF_2)) |
| 2,4-Decadienal | (**268**) |  |  |
| (*Z*)-2-Decenal | (**269**) |  |  |
| Dehydrodiosgenin | (**270**) | *S. babylonica* L*.* | ([González-Alamilla et al., 2019](#_ENREF_30)) |
| 1,3-Dioxane, 4-(hexadecyloxy)-2-pentadecyl | (**271**) | *S. babylonica* L. | ([Salem et al., 2011](#_ENREF_90)) |
| *p*-Dimethoxybenzene | (**272**) | *S. caprea* L. | ([Ahmed et al., 2017](#_ENREF_2)) |
|  |  | *S. aegyptiaca* L. | ([Karimi et al., 2011](#_ENREF_46)) |
| 2,5-Di-tert-butylphenol | (**273**) | *S. caprea* L. | ([Ahmed et al., 2017](#_ENREF_2)) |
| 2,6-Dimethylheptadecane | (**274**) | *S. alba* L. | ([Zarger et al., 2014](#_ENREF_117)) |
| 1-Dodecyne | (**275**) | *S. caprea* L. | ([Ahmed et al., 2017](#_ENREF_2)) |
| Eugenol | (**276**) | *S. aegyptiaca* L. | ([Karimi et al., 2011](#_ENREF_46)) |
| Metheleugenol | (**277**) |  |  |
| Ethyl *p*-ethoxybenzoate | (**278**) | *S. caprea* L. | ([Ahmed et al., 2017](#_ENREF_2)) |
| Ethylene glycol *m*-cresyl ether | (**279**) | *S. alba* L. | ([Zarger et al., 2014](#_ENREF_117)) |
| Geranyl acetone | (**280**) |  |  |
| Glycerol | (**281**) |  |  |
| 1-Heptadecyne | (**282**) | *S. caprea* L. | ([Ahmed et al., 2017](#_ENREF_2)) |
| Hexahydrofarnesylacetone | (**283**) |  |  |
| Hexanoic acid, 4-hexen-1-yl ester | (**284**) | *S. alba* L. | ([Zarger et al., 2014](#_ENREF_117)) |
| Hexatriacontane | (**285**) | *S. babylonica* L. | ([Salem et al., 2011](#_ENREF_90)) |
| 2-Hexyl-1-octanol | (**286**) | *S. caprea* L. | ([Ahmed et al., 2017](#_ENREF_2)) |
| 2-Hydroxy-6-methyl- benzaldehyde | (**287**) | *S. babylonica* L. | ([Salem et al., 2011](#_ENREF_90)) |
| (E)-4-(4-hydroxy-2,2,6-trimetyl-7-oxabicyclo  [4.1.0] heptan-1-yl)but-3-en-2-one | (**288**) | *S. babylonica* L. | ([González-Alamilla et al., 2019](#_ENREF_30)) |
| 3-Hydroxy-*β*-damascone | (**289**) | *S. alba* L. | ([Zarger et al., 2014](#_ENREF_117)) |
| Isomenthol | (**290**) |  |  |
| Isovanillic acid | (**291**) |  |  |
| Limonene oxide | (**292**) |  |  |
| 5-Methoxy-2-methylaniline | (**293**) |  |  |
| Menthyl acetate | (**294**) | *S. caprea* L. | ([Ahmed et al., 2017](#_ENREF_2)) |
| Methyl-12-methyltridecanoate | (**295**) | *S. babylonica* L. | ([Salem et al., 2011](#_ENREF_90)) |
| 2-Methoxy-4-vinylphenol | (**296**) |  |  |
| 4ˋ-Methoxyacetophenone | (**297**) | *S. aegyptiaca* L. | ([Karimi et al., 2011](#_ENREF_46)) |
| Myristicacid | (**298**) | *S. alba* L. | ([Zarger et al., 2014](#_ENREF_117)) |
| Nonadecane | (**299)** | *S. babylonica* L. | ([Salem et al., 2011](#_ENREF_90)) |
| 8-Nonenoic acid | (**300**) | *S. alba* L. | ([Zarger et al., 2014](#_ENREF_117)) |
| *cis*-2-Nonenal | (**301**) | *S. caprea* L. | ([Ahmed et al., 2017](#_ENREF_2)) |
| Octahydro-1-nitroso-1H-azonine | (**302**) | *S. alba* L. | ([Zarger et al., 2014](#_ENREF_117)) |
| Palmitic acid | (**303**) | *S. alba* L. | ([Zarger et al., 2014](#_ENREF_117)) |
|  |  | *S. tetrasperma* Roxb. | ([El-Shazly et al., 2012](#_ENREF_19)) |
| Palmitic acid methyl ester | (**304**) | *S. babylonica* L. | ([Salem et al., 2011](#_ENREF_90)) |
| 1-Pentadecyne | (**305**) | *S. caprea* L. | ([Ahmed et al., 2017](#_ENREF_2)) |
| 1-pentacontanol | (**306**) | *S. babylonica* L. | ([Salem et al., 2011](#_ENREF_90)) |
| Pentyl furan | (**307**) | *S. caprea* L. | ([Ahmed et al., 2017](#_ENREF_2)) |
| 10,12-Pentacosadiynoic acid | (**308**) | *S. alba* L. | ([Zarger et al., 2014](#_ENREF_117)) |
| Phenylethyl alcohol | (**309**) | *S. aegyptiaca* L. | ([Karimi et al., 2011](#_ENREF_46)) |
| 2-Propyl-tetrahydropyran-3-ol | (**310**) | *S. alba* L. | ([Zarger et al., 2014](#_ENREF_117)) |
| Rose oxide | (**311**) | *S. caprea* L. | ([Ahmed et al., 2017](#_ENREF_2)) |
| Succinic acid, monomethyl ester | (**312**) | *S. alba* L. | ([Zarger et al., 2014](#_ENREF_117)) |
| Stearic acid methyl ester | (**313**) | *S. babylonica* L. | ([Salem et al., 2011](#_ENREF_90)) |
| Stearyl aldehyde | (**314**) | *S. alba* L. | ([Zarger et al., 2014](#_ENREF_117)) |
| Stearic acid | (**315**) |  |  |
| 3,7,11,15-Tetramethyl-2-hexadecen-1-ol (phytol) | (**316**) | *S. babylonic* | ([Salem et al., 2011](#_ENREF_90)) |
| 1,2,3-Trichlorobenzene | (**317**) | *S. caprea* L. | ([Ahmed et al., 2017](#_ENREF_2)) |
| Tritetracontane | (**318**) | *S. babylonica* L. | ([Salem et al., 2011](#_ENREF_90)) |
| 2,5,8-Trimethyltetralin | (**319**) | *S. alba* L. | ([Zarger et al., 2014](#_ENREF_117)) |
| 2,5,5-Trimethyl-3-hexyn-2-ol | (**320**) |  |  |
| 3,4,4-Trimethyl-2-hexene | (**321**) |  |  |
| (E)-2-(2,2,6-trimethyl-7-oxabicyclo [4.1.0]heptan-1-yl) prop-1-en-1-ol | (**322**) | *S. babylonica* L. | ([González-Alamilla et al., 2019](#_ENREF_30)) |
